# Core–Shell Bottlebrush Polymers: Unmatched Delivery of Small Active Compounds Deep Into Tissues

**DOI:** 10.1002/smll.202408616

**Published:** 2024-12-16

**Authors:** Quoc Thang Phan, Jean‐Michel Rabanel, Dikran Mekhjian, Justine Saber, Araceli Garcia Ac, Hu Zhang, Victor Passos Gibson, Charlotte Zaouter, Pierre Hardy, Shunmoogum Aroonassala. Patten, Daria Boffito, Xavier Banquy

**Affiliations:** ^1^ Faculty of Pharmacy Université de Montréal 2940 Chemin de Polytechnique Montréal Québec H3T 1J4 Canada; ^2^ School of Pharmaceutical Sciences Faculty of Medicine University of Ottawa Roger Guindon Hall, 451 Smyth Rd Ottawa Ontario K1H 8M5 Canada; ^3^ Department of Pharmacology and Physiology Université de Montréal Montréal Québec H3T 1J4 Canada; ^4^ INRS Centre Armand‐Frappier Santé Biotechnologie 531, boul. des Prairies Québec Canada H7V 1B7; ^5^ Department of Chemical Engineering Polytechnique Montréal 2500 Chemin de Polytechnique Montréal Québec H3C 3A7 Canada; ^6^ Biomedical Engineering Institute Université de Montréal 2940 Chemin de Polytechnique Montréal Québec H3T 1J4 Canada; ^7^ Chemistry Department Faculty of Arts and Sciences Université de Montréal 2940 Chemin de Polytechnique Montréal Québec H3T 1J4 Canada

**Keywords:** bottlebrush polymer, drug delivery system, methacryloyloxyethyl phosphorylcholine, poly (D,L‐lactic acid), self‐assembly

## Abstract

The chemical structure of a delivery nanovehicle plays a pivotal role in determining the efficiency of drug delivery within the body. Leveraging the unique architecture of bottlebrush (BB) polymers—characterized by variations in backbone length, grafting density, and self‐assembly morphology—offers a novel approach to understanding the influence of structural properties on biological behavior. In this study, developed a drug delivery system based on core‐shell BB polymers synthesized using a “grafting‐from” strategy. Comprehensive characterization techniques, including nuclear magnetic resonance (NMR), gel permeation chromatography (GPC), and atomic force microscopy (AFM), employed to confirm the polymers’ structure. The BB polymers evaluated as carriers for molecules with differing hydrophobicity profiles, namely Rhodamine B and Paclitaxel. These nanocarriers systematically assessed for drug loading efficiency and penetration capabilities, compared to conventional polymeric micelles (PM) formed from linear amphiphilic polymers. BB‐based nanocarriers exhibited superior cellular uptake in both 2D and 3D cell culture models when compared to PM. Furthermore, analysis of drug distribution and particle penetration highlighted the profound influence of polymer morphology on biological interactions. These findings underscore the potential of unimolecular carriers with precisely defined structures as promising drug delivery platforms for a wide range of biomedical applications.

## Graphical abstract

The bottlebrush (BB) polymer‐ based drug unimolecular carriers with varying aspects synthesized by “grafting‐from” technique. The nanocarriers demonstrated superior performance in drug encapsulation efficiency and cellular uptake in both 2D and 3D cell cultures, compared to conventional polymeric micelles (PM). This suggests that the BB unimolecular carrier holds great promise as a drug nanocarrier for various biomedical applications.

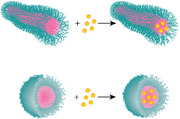

## 1 Introduction

Besides drug targeting, one of the most difficult challenges of drug delivery is to improve drug penetration in the diseased tissues.^[^ ^1^ ^]^ Drug nanocarriers are promising strategies to improve biodistribution,^[^ ^2^ ^]^ prolong blood circulation, and decrease toxicity,^[^ ^3^ ^]^ however they demonstrated limited diffusion in tumor, tissues, and across biological barriers. Many nano‐sized delivery agents including micelles,^[^ ^4^ ^]^ liposomes,^[^ ^5^ ^]^ polymeric nanohybrids,^[^ ^6^ ^]^ and inorganic nanoparticles^[^ ^7^ ^]^ have been explored to improve the pharmacological and therapeutic properties of emerging drugs. The molecular assemblies used as carriers are highly susceptible to the complex conditions of the body, such as temperature, ionic strength, pH, and the presence of serum proteins.^[^ ^8^, ^9^, ^10^, ^11^, ^12^ ^]^ This sensitivity often results in instability or premature dissociation of the self‐assembled carriers and off‐target release of drugs.

Bottlebrush polymers (BB) are branched polymers with polymeric side chains attached to a linear backbone. The unusual architectures of “bottlebrushes” provide several unique and potentially useful properties.^[^ ^13^, ^14^, ^15^ ^]^ When the density of side chains (i.e., the number of branches per molecule) decreases, the conformation of the polymers shows a transition from dense bottlebrush to loose comb. Moreover, varying the amphiphilicity balance of the sidechains enables BB to generate diverse assemblies by varying the length ratio of hydrophobic and hydrophilic domains in the sidechains.^[^ ^16^ ^]^

A growing body of evidence from recent studies has demonstrated that functionalized BB polymers, which comprise polymeric backbone decorated with long pendent chains, constitute an innovative and effective platform for drug delivery systems.^[^ ^17^, ^18^, ^19^ ^]^ Furthermore, BB polymers have many advantages compared to more traditional polymeric materials, such as linear polymers, to prepare drug nanocarriers. For instance, they display a remarkably long circulation time, low opsonization, high diffusion coefficient in dense or confined media, and high drug loading.^[^ ^20^ ^]^ In light of these advantages, the development of an efficient BB delivery platform capable of penetrating deeply dense tissues such as hard‐to‐treat solid tumors is a promising avenue for further research.^[^ ^21^ ^]^ Solid tumors are particularly difficult to treat using conventional chemotherapy since they require high doses of chemotherapeutic which can generate harmful toxicity effects and limit treatment efficacy. Another issue limiting drug efficacy is the low diffusivity of drug into the dense tumor microenvironment.

To develop core–shell BB polymers, a hydrophobic core composed of poly‐D,L‐lactic acid (PLA), and poly(2‐methacryloyloxyethyl phosphorylcholine) (MPC) as hydrophilic shell was used. PLA is a polymer that has been one of the most extensively utilized polymers in the biomedical realm owing to its biocompatibility, biodegradability, customizable characteristics, and established formulations. PLA, used as a core‐forming segment in block copolymers sidechains, facilitates polymer self‐assembly in aqueous environments due to its hydrophobic nature.^[^ ^22^ ^]^ MPC, on the other hand, is a hydrophilic polymer and has been demonstrated to provide water solubility and resistance to protein adsorption, enhancing the stability and pharmacokinetic profiles of copolymer‐based nanoparticles.^[^ ^23^ ^]^ The unimolecular structure of the BB polymer delivery system could enable it to protect the therapeutic payload from rapid elimination, to reach deep regions of the tumor, and to deliver its payload in a controlled manner.^[^ ^24^ ^]^ Additionally, with its unimolecular structure, BB presents itself as a promising alternative to micellar systems whose capacity for loading drugs is compromised as their sizes cannot be effectively controlled at high loading content.^[^ ^25^ ^]^ While certain conjugation techniques have been employed to load drugs into polymeric nanocarriers such as BB polymers, they often face limitations imposed by the chemical structure of the drugs that require the chemical modifications. In contrast, encapsulation in core–shell BB polymers offers the advantage of simple and rapid preparation without altering the drug's structure through physical encapsulation by electrostatic or hydrophobic interactions.^[^ ^26^ ^]^

This study presents the first synthesis, characterization, and evaluation of core–shell BB for drug delivery application. The aim was to develop a drug delivery vehicle based on BB polymers that would enhance the penetration of the drug into solid tumors and significantly improve its therapeutic efficacy. The pendant chain grafting density of the BB polymer was varied to evaluate the diffusion and penetration of the macromolecular carrier into the tumors in vitro as well as for their biodistribution in vivo. The designed unimolecular BB polymers displayed a high proportion of PLA segments which can encapsulate large amounts of hydrophobic drug through hydrophobic interaction compared to a linear diblock polymer of similar composition assembled into a spherical particle. To investigate the significance of BB polymer loaded with anticancer drugs in terms of antitumoral efficacy and deeper tissue penetration ability, the cellular uptake and cytotoxicity tests were performed in both 2D and 3D cell models.

## 2 Results and Discussion

### 2.1 Synthesis of Core–Shell BBs with Different Grafting Densities

**Scheme 1:**
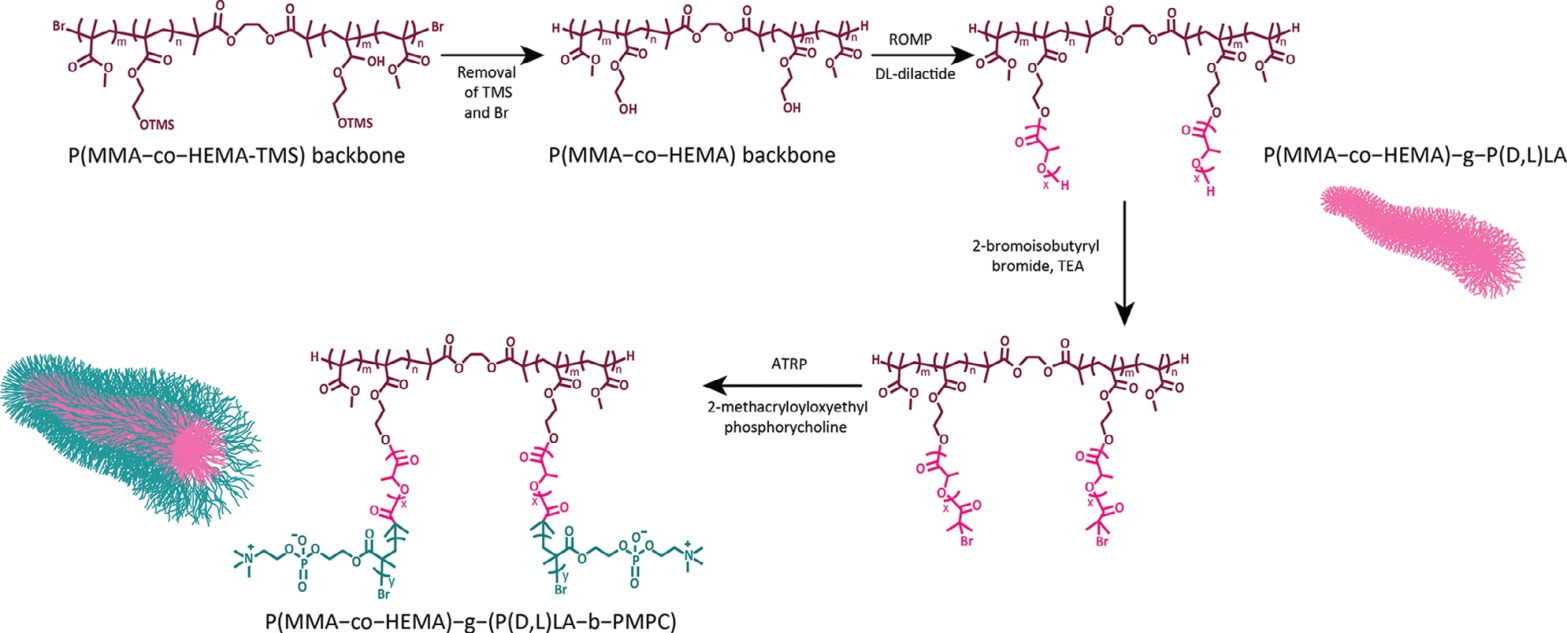
Synthetic pathway of core–shell bottlebrush polymers with P(D,L)LA core – PMPC as an outer shell.

To prepare the BB with core–shell structure with amphiphilic side‐chain, we designed a synthetic polymer that comprised of: 1) A polymeric backbone based on poly(HEMA‐TMS)‐co‐MMA with different molar ratios of HEMA‐TMS/MMA, to later evaluate the effect of side chains grafting density, and 2) Amphiphilic side chains, with PLA as hydrophobic block, and MPC as the hydrophilic block. The synthesis of the core–shell BB polymers was obtained in five steps via the combination of atom transfer radical polymerization (ATRP), ring‐opening polymerization (ROP), and post‐modification techniques (**Scheme** **1**). And the successful synthesis of core–shell BBs was confirmed by NMR and GPC characterizations.^[^ ^27^ ^]^ For each step of the polymerization, the obtained conversion ratios and molecular weights were summarized in Table S1 (Supporting Information).

The backbone was synthesized through the copolymerization of (2‐trimethylsiloxy) ethyl methacrylate (HEMA‐TMS) and methyl methacrylate (MMA), with a molar ratio ranging from 30% to 72%, It was subsequently employed as a macroinitiator for the side chains. The kinetics of backbone polymerization were followed by NMR (Figure S1, Supporting Information), and the composition of HEMA‐TMS and MMA copolymer was found to be very similar to the monomer feed ratios in all cases.^[^ ^28^ ^]^ Subsequently, the conversion ratios of the backbone were controlled for specific lengths of BB polymers, in accordance with the obtained kinetics.

The side chains grafting density was obtained from NMR proton spectrum by comparing the integral of ‐OTMS groups at 0.0 ppm to ‐CH_3_ groups of MMA at 3.4 ppm (Figure S2, Supporting Information). The targeted degree of polymerization (DP) of the polymer backbone was ≈1000, which corresponds to a contour length of ≈150 nm. In the next step, OTMS moieties were removed to produce the hydroxyl groups on the backbone which are used to initiate the ROP of the di‐lactide. As can be seen in the NMR proton spectrum (Figure S3, Supporting Information), the OTMS peak at 0.0 ppm completely disappeared and a new peak corresponding to the hydroxyl groups (‐OH) at 4.8 ppm appeared indicating the successful hydrolysis of TMS. Next, the hydrophobic block (PLA) was synthesized using ROP with DBU as the catalyst. The DP of the PLA block was maintained below 20 units to ensure that the BB structure would not collapse by the inter‐hydrophobic interaction when immersed in water. The number of lactic acid units in the side chains was calculated by proton NMR by comparing the integral of ‐CH_3_ on the backbone at 3.6 ppm to ‐CH of PLA sidechain at 5.3 ppm (Figure S4, Supporting Information). BB polymers are unimolecular structures. By adjusting the degree of polymerization (DP) of the backbone, BB polymers provide an effective way to create molecules with tightly packed side chains of various compositions, such as hydrophobic, hydrophilic, and amphiphilic groups, suitable for encapsulating different types of molecules.^[^ ^29^, ^30^ ^]^

The GPC traces of the polymeric backbone before and after the grafting of PLA are also shown in Figure S6 (Supporting Information). The backbone with different numbers of HEMA‐TMS showed a low dispersity, Đ_M_ = M_w_/M_n_ = 1.08; 1.11; and 1.05 for a grafting density G = 0.72; G = 0.5; and G = 0.3, respectively. For the grafting of PLA, due to the rapid reaction (3mins), the GPC traces showed a relatively low dispersity Đ_M_ = 1.13; 1.2; and 1.21 for PLA grafted backbone with G = 0.72; G = 0.5; and G = 0.3, respectively. Overall, the calculated molecular weight by NMR and the Mn obtained by GPC were similar for the three different BB polymers as shown in Figures S3 and S5 (Supporting Information), respectively. Subsequently, the end groups of the PLA sidechain block were functionalized with a bromide moiety to prepare the ATRP macroinitiator as described before.^[^ ^31^, ^32^, ^33^ ^]^ The full substitution of the bromide groups was confirmed by NMR by comparing the integral of the ‐CH_3_ group (at 3.6 ppm) on the MMA in the backbone and the signal at 1.9 ppm, attributed to the methyl moieties of the OOC‐C(CH_3_)_2_Br ester groups, appearing after the esterification reaction (Figure S5. Supporting Information).

With this macroinitiator, the growth of polyzwitterionic (PMPC) side chain extension was performed through the “grafting from” approach. PMPC was chosen due to the superhydrophilicity of this polymer as well as recent reports showing that surface grafted PMPC brushes exhibit extremely good biocompatibility and anti‐fouling properties.^[^ ^34^, ^35^, ^36^ ^]^

Varying the grating density of the side chains in the BB polymer offers insights into its effect on drug loading capacity, release, and tissue penetration in vitro and in vivo of the core–shell BB polymer.^[^ ^37^ ^]^ The composition of synthesized polymers is summarized in **Table** **1**.

**Table 1:** Composition and characteristics of BBs polymer with different backbone and pendant side chains.

No.	Formulation	DP of Backbone	Grafting density [G]	PMPC/PLA molar ratio, [R]	f [w%]^a)^	Mn of core–shell BB [g/mol]^b)^	Brush size, nm [Length × Width]^c)^	Core thickness, nm^c)^
BB1	(HEMA_800_‐co‐MMA_300_)‐g‐(P(D,L)LA_14_‐b‐MPC_140_)	1100	0.72	10	2.76	35161708	138.3 **×** 76.1	13.8 ± 3.0
BB2	(HEMA_600_‐co‐MMA_600_)‐g‐(P(D,L)LA_17_‐b‐MPC_150_)	1200	0.5	8.82	3.16	38555026	134.6 **×** 88.2	33.5 ± 0.8
BB3	(HEMA_240_‐b‐MMA_560_)‐g‐(P(D,L)LA_22_‐b‐MPC_180_)	800	0.3	8.18	3.57	13608448	117.5 **×** 83.3	32.8 ± 0.74
PM	P(D,L)LA_21_‐b‐MPC_135_	–	–	–	4.03	43849	–	41.2 ± 1.6

^a)^ “f” represents the hydrophobic weight fraction of PLA and polymeric backbone in the polymer;

^b)^ Mn: the molecular weight was determined by NMR. The number of HEMA, PLA, and MPC were calculated based on NMR spectra;

^c)^ Brush size and core thickness were calculated based on the AFM images.

Because of the amphiphilicity of the synthesized diblock and BB polymers, their behavior in aqueous solution was initially evaluated to assess their self‐assembly capability as a function of polymer concentration. A series of polymer solutions at varying concentrations, ranging from 100 to 10 µg mL^−1^, were prepared (Figure S7, Supporting Information) and deposited on mica surfaces for AFM imaging. This imaging enabled the assessment of particle formation and morphology with sub‐molecular resolution. At a high polymer concentration (100 µg mL^−1^), BB polymer aggregates up to 1000 nm in size were observed across all grafting densities of the BB polymer.^[^ ^16^ ^]^ For the dilution at 10µg mL^−1^, the aggregate structures disappeared, revealing single cylindrical brush molecules. Due to the difference of grafting density (G = 0.3, G = 0.5, and G = 0.72), BB polymers exhibited a rod‐like morphology with some differences in their shape and their contour length as summarized in Table 1. In addition to the AFM images, the critical micelle concentration of BB polymers was also determined using curcumin as a fluorescent probe, as previously outlined in our research. Moreover, our team has recently demonstrated that core–shell BB polymers are capable of self‐assembling into large aggregates.^[^ ^16^, ^38^, ^39^ ^]^ Since we aim to compare the BB polymers and micelles formed from linear diblock polymers as drug delivery systems, the BB polymers were formulated at low concentrations where they exist as single molecules rather than large aggregates.

### 2.2 Encapsulation and Release Kinetics of Model Drugs

The amphiphilic nature of the core–shell BB polymer together leads to the hypothesis that these polymers could serve as versatile nanocarriers for small active molecules.^[^ ^40^ ^]^

**Figure 1:**
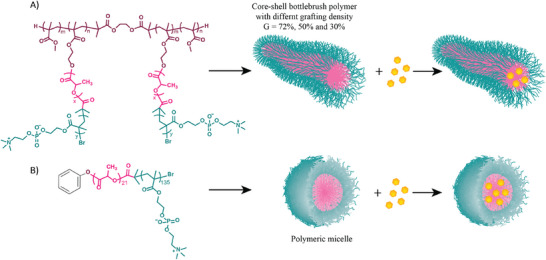
Schematic illustration of A) BB polymers, and B) Self‐assembled polymeric micelles (PM) of linear diblock polymer as a platform for drug encapsulation.

The fluorescent dye Rhodamine B (RhB) and the chemotherapeutic agent Paclitaxel (PTX) were used to investigate the effects of the BBs' unimolecular structure on drug encapsulation, compared to micelles prepared from diblock linear polymers (**Figure** **1**). With log P values of 1.9 and 3.6 respectively, RhB and PTX were chosen to confirm the BB polymers' capability to load different hydrophobic drugs. After verifying the morphology of the BB polymers' unimolecular structure and micelles via AFM, drug loading/release was determined using HPLC (Figure S8, Supporting Information).

**Figure 2:**
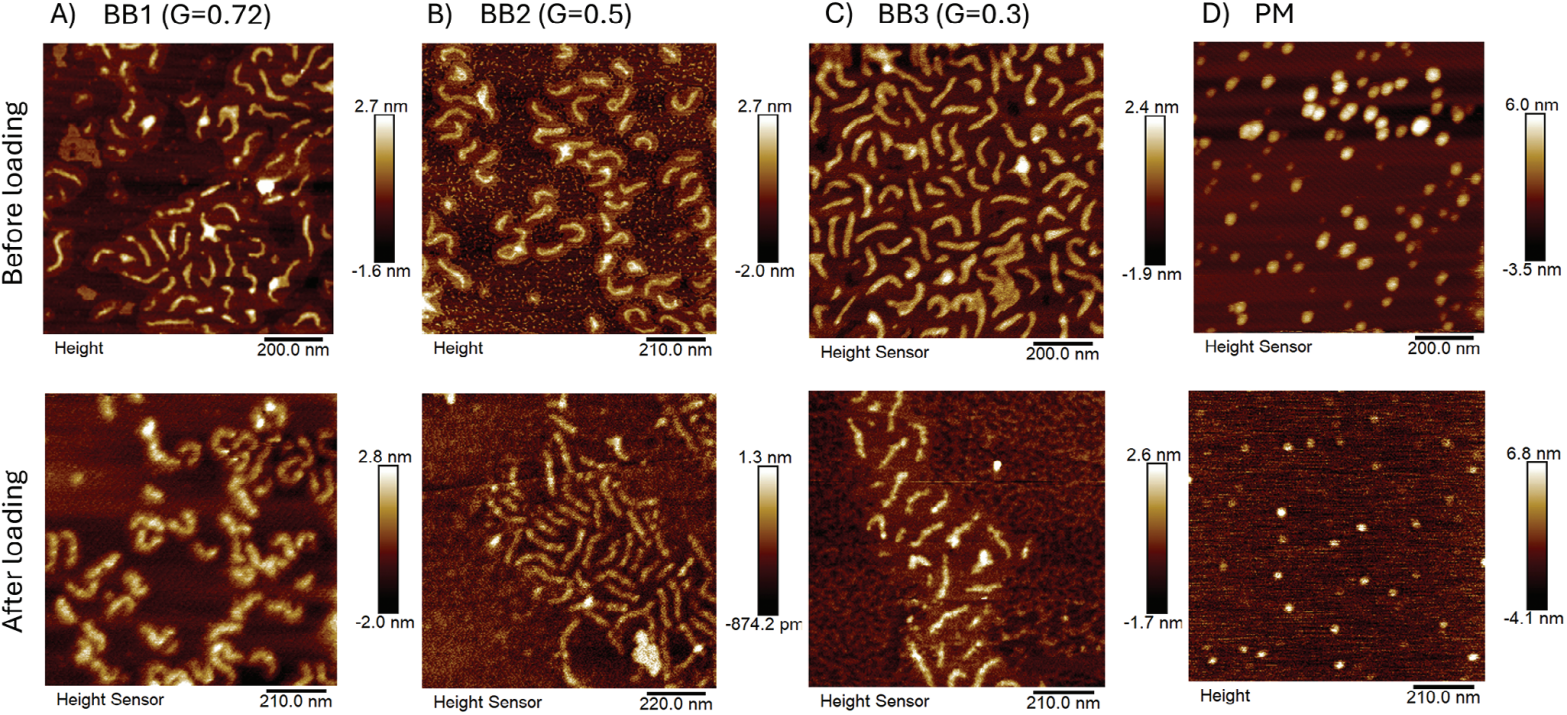
Morphology of BBs polymers before and after the loading of PTX; A) BB1 (G = 0.72); B) BB2 (G = 0.5); C) BB3 (G = 0.3) and D) PM. The upper panels are AFM images of BB polymers before loading and the lower panels are corresponding AFM images after the loading.

Atomic force microscopy imaging (AFM) of the drug‐loaded BB polymers revealed that the dimensions of PTX‐loaded worm‐like BB polymers remained unaltered (see **Figure** **2**). Following the drug‐loading process, the BB polymers retained their original morphology with no sign of aggregation, exhibiting only a slight increase in core thickness which can be attributed to the incorporation of molecules into the core of BB polymers. Furthermore, due to this encapsulation, the BBs exhibited slight collapse, resulting in changes in curvature and increased core density.

Before loading of PTX, the thickness of BB1, BB2, and BB3 was 13.8 ± 3.0, 33.5 ± 0.8, and 32.8 ± 0.7 nm, respectively. After loading, these values were increased slightly because of the presence of PTX in the core of the polymer (21.7 ± 1.3 nm for BB1, 35.2 ± 5.9 nm for BB2, and 37.6 ± 3.7 nm for BB3). In the case of the PM, the size of the micelles was reduced from 41.2 ± 1.6 to 23.1 ± 0.9 nm after loading.

The data is in agreement with DLS measurements. The determined sizes of blank and PTX loaded BB1, BB2, and BB3 polymers were not significantly different in DLS. For PM, we determined the sizes of blank and drug‐loaded PM to be respectively 41.2 ± 1.6 and 23.1 ± 0.9 nm by AFM. Size obtained by DLS were respectively 51.7 and 32.4 nm for blank and drug‐loaded PM (Figure S9, Supporting Information). The reduction in the size of the drug‐loaded micelle was linked to the neutralization and condensation of hydrophobic poly(lactic acid) units upon the incorporation of the lipophilic molecule – PTX.^[^ ^41^, ^42^ ^]^

**Figure 3:**
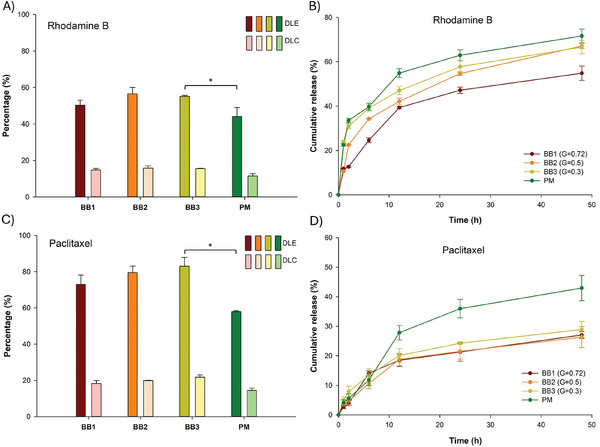
Encapsulation efficiency of A) Rhodamine B and C) Paclitaxel in core–shell BB polymers compared to polymeric micelles; Release profiles from BBs polymer and polymeric micelles of B) Rhodamine B and D) Paclitaxel (* *p* < 0.05, *n* = 3).

At the same weight feeding ratio (polymer: drug = 3:1), all the BB polymeric micelles were able to encapsulate the two molecules tested regardless of their hydrophobicity. For the lower hydrophobicity molecule – RhB (**Figure** **3**A), drug loading efficiency (DLE) of the different BB polymers tested varied from 50% to 55%, and the drug encapsulation capacity (DEC) varied from 14% to 15% with no significant difference between BB polymers. However, in the case of RhB‐loaded polymeric micelles, the DLE was 44% while the DLC was 11%. The more hydrophobic molecule, PTX (log P = 3.6) was also successfully loaded into the BB polymers at a higher efficiency compared to polymeric micelles (Figure 3C). The highest loading efficiency of PTX was observed with BB3 (83%) while the lowest was with BB1 (73%) and the DLCs were 21.7% and 18.5%, respectively. The linear diblock polymer showed DLE and DLC of 57% and 14.4% respectively. DLE and DLC were thus ≈20% and 7% lower for the polymeric micelles compared to the BB polymers.

Moreover, to assess the encapsulation of drugs into polymer formulations, we conducted a nanoflow cytometry experiment to characterize the Cy5 encapsulation within the BB3 and PM particles (Figure S10, Supporting Information).

Nano‐flow cytometry is an advanced analytical technique that combines flow cytometry with nanoparticle analysis, enabling high‐resolution characterization of individual nanoparticles (NPs) at the single‐particle level. It uses light scattering and fluorescence detection to measure particle size, concentration, and surface markers. This technique is particularly useful for assessing the encapsulation efficiency of fluorescent dyes like Cy5 within nanoparticles, allowing for precise quantification of loading levels.

As illustrated in Figure S10A,D (Supporting Information), the presence of side scatter (SS) peaks is apparent for the empty nano. Blank nanoformulations exhibited no fluorescence signal as expected. However, for the Cy5‐loaded nanoformulations – as illustrated in Figure S10B,E (Supporting Information), the particles detected by the SS signal exhibit simultaneous fluorescence, indicating that both BB3 and PM are fluorescent. The level of fluorescence is proportional to particle size as expected, in Figure S10C,F (Supporting Information), overlays of the two populations (fluorescent and non‐fluorescent) are displayed. This confirms the efficient encapsulation of Cy5 in the BB polymers and in the PM.

The differences in encapsulation efficiencies could be explained by the differences in volumes of the hydrophobic cores of the different structures. In the case of the polymeric micelle, the number of hydrophobic segments (P(D,L)LA) is much smaller compared to BB polymers. As a result, the relatively high volume of the hydrophobic domain of BB polymers contributes to an increase in the DLC and DLE via hydrophobic interactions with loaded molecules.^[^ ^43^ ^]^ As a self‐assembled system, undesired leaking and premature drug release of molecules from PM could be checked by the FRET experiment.^[^ ^44^ ^]^

Furthermore, from the loading results of RhB and PTX it can be observed that the BB polymer with small grafting density (BB3, G = 0.3) shows relatively higher DLE and DLC compared to the other two BB polymers (BB1 and BB2). This difference suggests that to achieve high encapsulating capacity in the core–shell BB polymers, side chains grafting density must be tuned adequately. In case the grafting density of side chains is too high, it prevents the insertion of molecules into the hydrophobic pocket of the carrier and reduces the DLE and DLC of hydrophobic agents such as PTX.^[^ ^45^ ^]^

Quantitative analysis of the release of RhB and PTX from polymers was conducted in PBS solution, incubated at physiological pH (7.4) at 37 °C. The RhB loaded in smaller grafting densities BB (G = 0.5 and G = 0.3) and polymeric micelles, had faster release profiles within 48 h compared to the high grafting density BB1 (G = 0.72) (Figure 3B). For micelles and BB polymers with G = 0.5 and 0.3, approximately 35% of the rhodamine B was released within the first 6 h. By comparison, for BB polymer with G = 0.72, only 22% of the RhB was released within the same period. Similarly, after 48 h, only 54.8% of the RhB was released from BB1, while in the same period, the release of RhB from micelles, BB2, and BB1 were 71.4%, 66.7%, and 67.1%, respectively. As illustrated in Figure 3D, PTX molecules were gradually released from micelles with a strong burst release during the first 12 h, to reach 42.9% of released drug at 48 h. On the other hand, the three BB polymers showed a sustained release of over 48 h without rapid release. After 48 h, the release of BB polymers remained at 23.6% – about 20% lower than the drug release from micelles. It is worth mentioning that the quantity of PTX indicated in Figure 3D may have been underestimated compared to the actual amount being released. The release of PTX after 48h from all formulations was below 50% likely due to PTX susceptibility to degradation over time at neutral pH. PTX release can also be slowed down by the formation of crystals due to its low solubility in PBS.^[^ ^11^, ^46^, ^47^ ^]^

When comparing the release profiles of the different formulations, both encapsulated molecules in BB polymers exhibited a slower release when compared to polymeric micelles. This could be explained by different factors such as the fact that polymeric micelles structure is known to be dynamic and can therefore dissociate partially in the medium leading to a stronger burst release of the entrapped molecules.^[^ ^48^ ^]^ On the other hand, the unimolecular structure of the core–shell BB polymers offers a stable environment that prevents the rapid release of the drug from the core. The BB polymer with the highest grafting density exhibits a lower loading capacity but maintains a slower release rate. It seems that the higher grafting density of the side chains restricts the accommodation of active molecules within the core of the BB polymer, while also providing a tighter and more compact environment, which collectively helps to reduce molecular diffusion and escape.^[^ ^25^, ^37^ ^]^ In addition, the release kinetics of BB polymers could be influenced by their biodegradability in physiological environments. This was investigated using AFM images and DLS measurements in a previous report. After a prolonged incubation period, the side chains of the BB polymer underwent degradation due to the hydrolysis of ester groups, resulting in the loss of the cylindrical brush structure into smaller fragments.^[^ ^16^ ^]^

### 2.3 Cytotoxicity and Cellular Uptake of BBs Polymer on 2D Cell Lines

In this section, three different cell lines were used to evaluate the cytotoxicity of the nanocarriers. The first cell line, hepatocyte carcinoma (HepG2), was selected as a model to test hepatic toxicity. The liver plays a crucial role in clearing nanomaterials from the bloodstream, making hepatocyte sensitivity to nanomaterial‐induced toxicity a key consideration in developing an innovative drug nanocarrier. The other cell lines considered were human glioblastoma (U87) and human colorectal adenocarcinoma (Caco‐2). These cell lines represent hard‐to‐treat solid tumors with natural defense mechanisms that resist chemotherapy, making them effective models for testing the safety of our chemotherapy delivery strategies.^[^ ^49^, ^50^ ^]^

**Figure 4:**
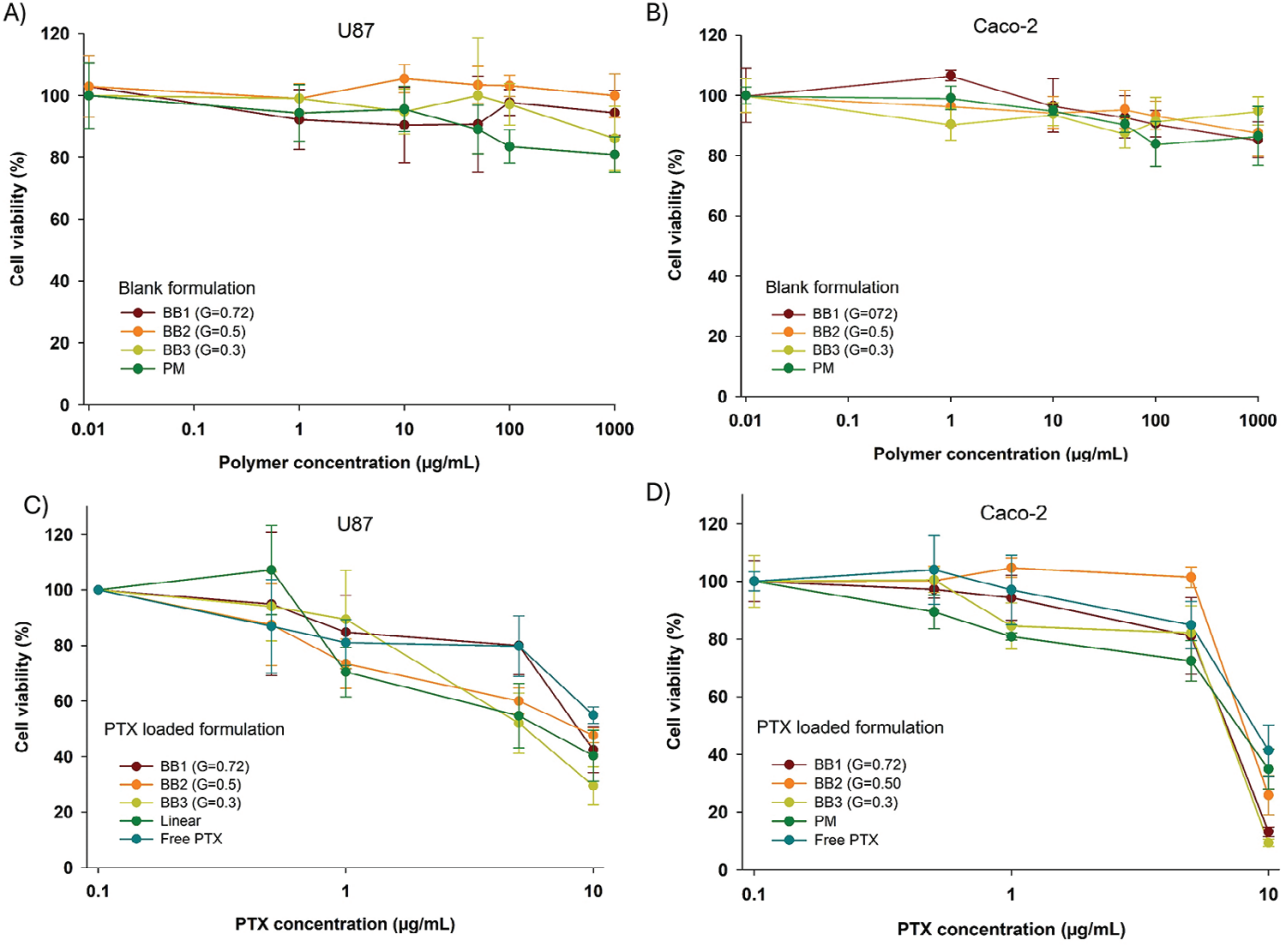
Cells viability was determined with A) U87; and B) Caco‐2 cells after 72 h incubation with different concentrations of blank BB polymers and polymeric micelles. 72‐h antiproliferation study of PTX loaded BB and linear polymers on C) U87; and D) Caco‐2 cells at different concentrations.

Blank formulations (without drug) were incubated at various concentrations for 72 h, after which U87 (**Figure** **4**A), Caco‐2 (Figure 4B), and HepG2 (Figure S11, Supporting Information) cells were analyzed using the MTS assay. The results of the biocompatibility assays indicated that even at the highest concentration tested (1000 µg mL^−1^), the blank BB polymers and polymeric micelles did not induce any signs of cell death. The in vitro cytotoxicity of PTX‐loaded formulations compared to free PTX was also evaluated using MTS assays on U87 and Caco‐2 cells (Figure 4C,D). It was found that both PTX‐loaded polymers and free PTX exhibited significant cytotoxicity, with cell viability decreasing as PTX concentrations increased. In both cell lines, at a low concentration of 0.5 µg mL^−1^, PTX‐loaded BB polymers and polymeric micelles showed similar levels of cell viability compared to free PTX (Figure 4C,D). This can be attributed to the slow release of the drug from the BB polymers and polymeric micelles. However, at higher concentrations (10 µg mL^−1^), PTX‐loaded polymers demonstrated higher efficacy in killing U87 and Caco‐2 cells compared to free PTX. This increased efficacy is likely due to the nanocarriers' ability to facilitate drug entry into the cells, whereas free PTX uptake is limited due to its poor solubility in the medium. Among the three BB polymer nanocarriers, there was no significant difference in therapeutic efficacy. However, compared to polymeric micelles, BB polymers reduced the release of PTX at low concentrations (0.1–5 µg mL^−1^), leading to a slower rate of cell death. This slower drug release effect is presumably due to the presence of large hydrophobic domains within the BB polymers, as discussed earlier. The therapeutic efficacy of PTX‐loaded polymers against Caco‐2 cells 2D model was further visualized by microscopy in bright field mode (Figure S12, Supporting Information). At high concentration (10µg mL^−1^), the treatment of PTX‐loaded polymers led to nearly complete cell death, with a large number of dark cells on the plate well surface. Additionally, the appearance of some needle‐shaped crystals was evident in some cases, which indicates the release of PTX from the carrier and its recrystallization in the cell culture medium due to its low solubility. The MTS assay and microscope images indicated that the anticancer efficacy results observed for the three BB nanocarriers were consistent with the increase in PTX concentration.

**Figure 5:**
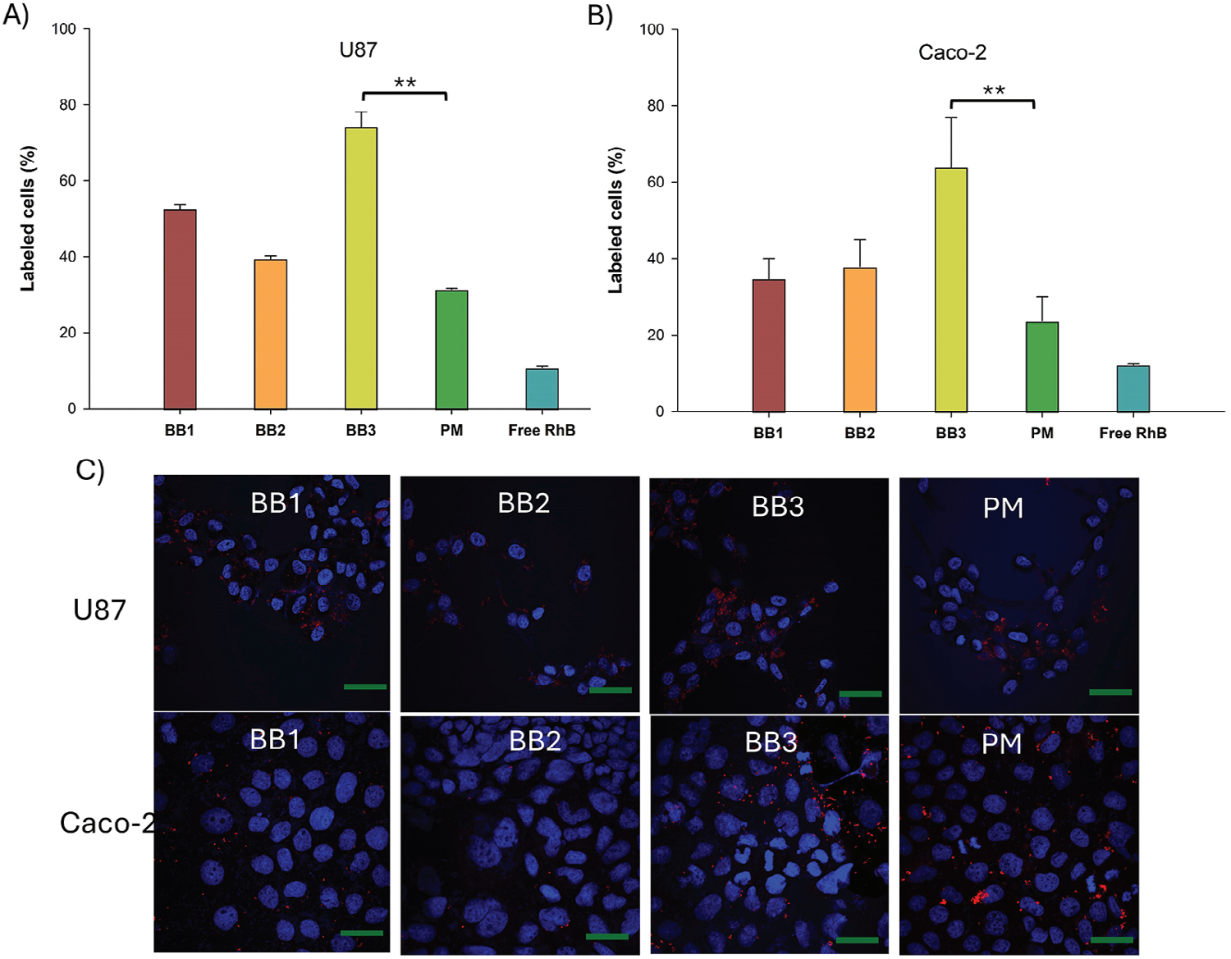
Cellular uptake of Rhodamine B and Rhodamine B encapsulated in BB and polymeric micelles on 2D cell cultures. Upper panel: Flow cytometry analysis of A) U87; and B) Caco‐2 treated with free RhB and RhB encapsulated in BB polymers and polymeric micelles. Lower panel: c) CLSM images of U87; and Caco‐2 cancer cell monolayers after 24 h incubation with the different formulations. Red channel = RhB‐loaded nanocarrier or Free RhB; Blue channel: Cell nucleus. Scale bar: 100 µm (** *p* < 0.01).

Cell uptake efficiency of RhB‐loaded BB polymers and polymeric micelles in Caco‐2 and U87 cells was quantified by flow cytometry (**Figure** **5**A,B). For the BB polymers, the smallest grafting density BB3 (G = 0.3) exhibited slightly higher uptake efficiency (71% cell labeling in U87 cells and 63% cell labeling in Caco‐2 cells) compared with BB1 (G = 0.72) and BB2 (G = 0.5). Moreover, compared to free RhB and RhB‐loaded polymeric micelles, BB3 showed superior cell uptake, supporting the notion that cell penetration of BB unimolecular particles was more effective than polymeric micelles cell uptake.

To assess the uptake of BB polymers and polymeric micelles by Caco‐2 and U87 cells, the internalization and intracellular distribution of RhB‐loaded nanocarriers were monitored using confocal microscopy (Figure 5C). The images confirmed the trend observed in the flow cytometry results. After 12 h of incubation, a strong red fluorescence signal was detected in both cell lines for BB3 (G = 0.3), indicating faster internalization of BB polymers with optimal grafting density. The cylindrical structure of BB3 not only provides a larger surface area for endocytosis compared to PM, but the lower grafting density also lowers the bending energy required for cellular internalization.^[^ ^51^, ^52^, ^53^ ^]^ The control experiments conducted with free RhB demonstrated that free RhB alone was unable to be taken up effectively by both cell lines (Figures S13 and S14, Supporting Information). This result also indicated that the uptake of free RhB into cells following premature RhB release or carrier disruption in the cell culture medium could be ruled out.

This finding aligns with a study by Robert et al.,^[^ ^54^ ^]^ which showed that spherical shapes may not be ideal for promoting internalization, while prolate cylinders appear to be more efficient. Cylindrical particles, with a larger volume compared to spherical particles of the same diameter, tend to exhibit faster uptake kinetics. Additionally, Liping et al.^[^ ^55^ ^]^ noted that endocytosing ellipsoidal particles is more challenging than spherical nanoparticles due to their greater curvature, which requires more membrane bending energy. These factors help explain why BB3 demonstrates better cellular uptake compared to the other two BB polymers and PM.

### 2.4 Tumor Penetration and Therapeutic Efficacy on 3D Tumor Spheroid

**Figure 6:**
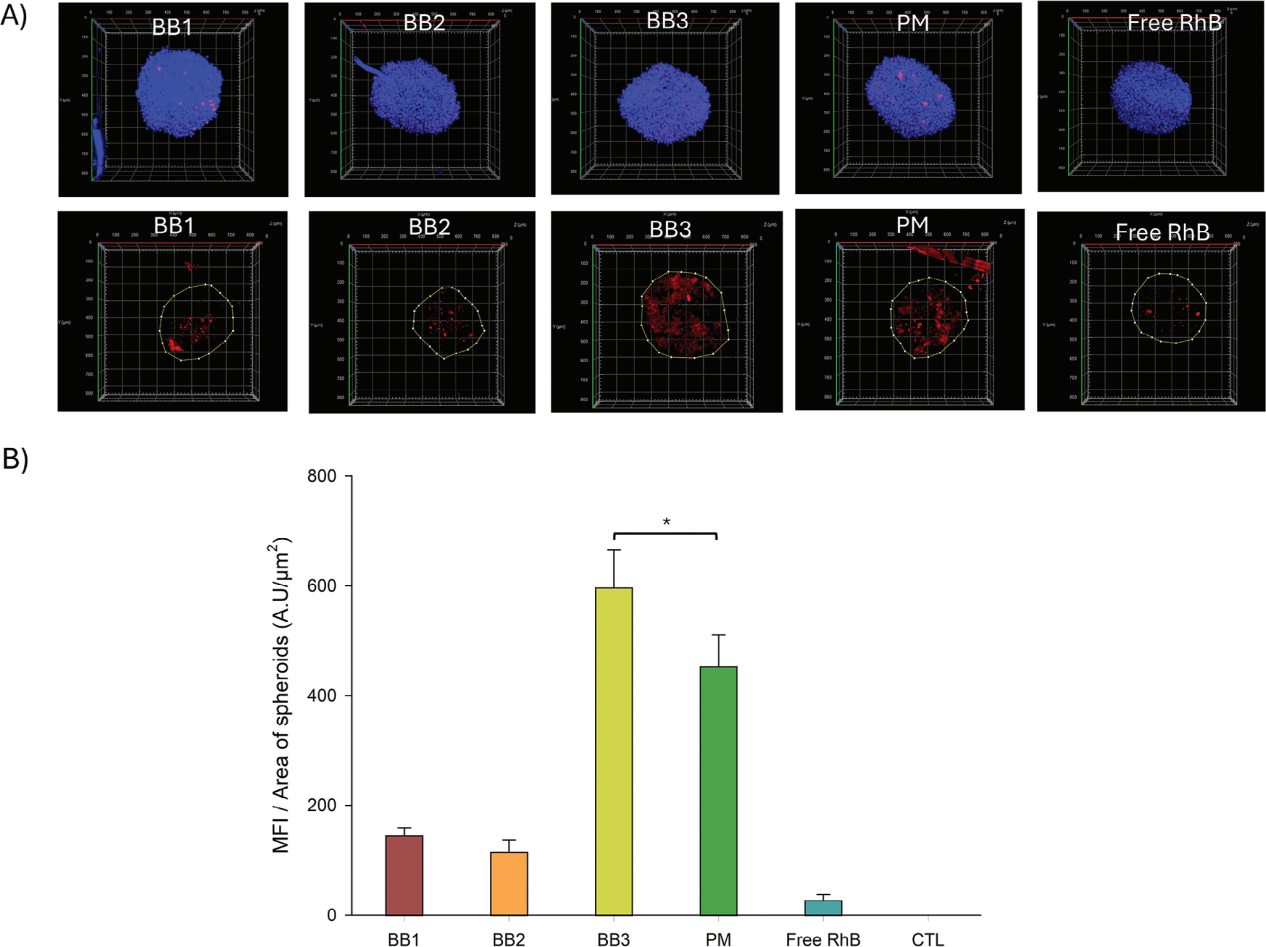
Tumor penetration efficacy of Free RhB and RhB encapsulated in BB polymers and polymeric micelles. A) Full projection of U87 spheroids grown for 24 h incubation (nuclear staining, upper panel) and equatorial projection (RhB channel, lower panel). Box edge length is 800µm. B) Mean fluorescence intensity (MFI) of RhB per unit area of spheroid (maximal projection) compared to free RhB, and RhB loaded polymers. Red channel: Rhodamine B; Blue channel: cell nucleus (* *p* < 0.05, *n* = 5).

To assess the impact of nanocarriers’ shape on their ability to penetrate tumors, 3D spheroids based on U87 cells were prepared using ultra‐low adhesion plates. The spheroids were incubated for 4 h with different RhB‐loaded nanoformulations before imaging. Confocal laser scanning microscopy (**Figure** **6**A) revealed that the fluorescence intensity of RhB from BB3‐treated spheroids was higher compared to those treated with polymeric micelles, BB1, and BB2. The confocal images showed the presence of the nanocarriers not only at the periphery of the tumor but also in its inner regions. Figure 6A indicates that BB3 penetrated deeply into the spheroids, likely due to its flexible structure and large aspect ratio, allowing it to navigate more efficiently into confined spaces such as the spheroid core. Quantitative analysis of RhB intensity per unit area of the spheroid (largest projection, Figure 6C) confirmed that the highest amount of fluorophore inside the spheroids was observed for BB3 compared to the other formulations.

Echoing the results observed in 2D cell cultures, BB3 demonstrated significant penetration and diffusion within 3D multicellular spheroids. As previously shown, BB polymers capitalize on their flexibility and pronounced shape asymmetry to diffuse efficiently in crowded environments through reputation diffusion rather than Brownian motion.^[^ ^56^, ^57^ ^]^ Earlier data revealed that BB polymers can successfully escape the bloodstream, cross biological barriers like the blood–brain barrier, and diffuse into dense tissues such as the brain. Additionally, polymers with more flexible and numerous side chains tend to exhibit slower diffusion into the spheroid core compared to those with fewer side chains.^[^ ^39^ ^]^

We observed a significant association of BB polymers with cancer cells at a specific grafting density, as indicated by the high fluorescence intensity in both 2D and 3D models. This penetration behavior of BB3 is consistent with findings from other studies. Similar to the results reported by Parathan et al.,^[^ ^24^ ^]^ they found that increasing the grafting density of the hydrophobic domain led to greater association of BBs with cells. However, when the grafting density exceeded 60%, the fluorescence intensity was reduced to half that of linear polymers, suggesting diminished cell association beyond a certain level of hydrophobicity. Additionally, Huaan et al.^[^ ^25^ ^]^ demonstrated that BB polymers with an optimal number of side chains achieved superior penetration into MCF‐7 spheroids compared to spherical particles, as quantified in their 3D model.

**Figure 7:**
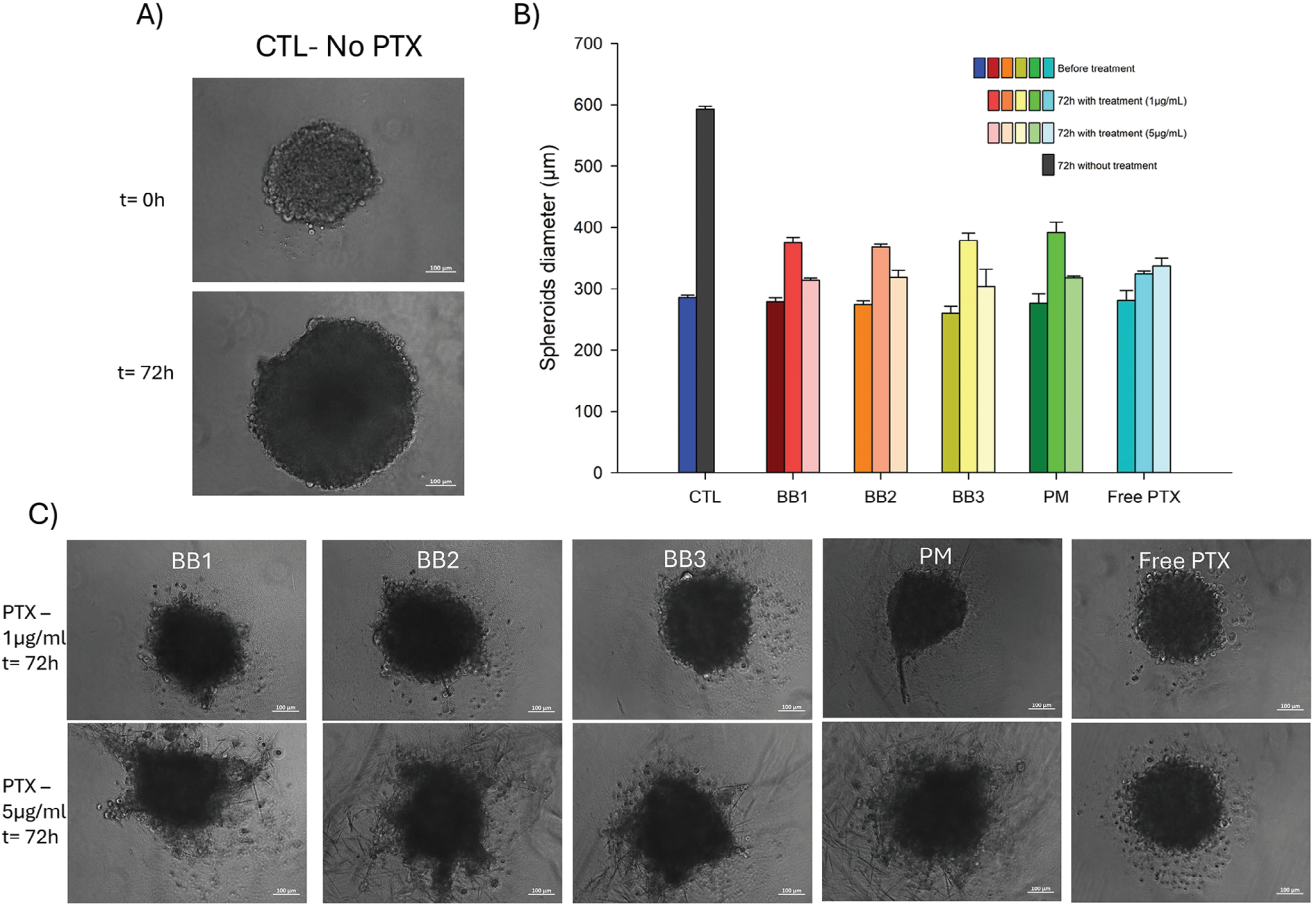
Efficacy study of free PTX and PTX encapsulated in BB polymers and polymeric micelles on 3D Spheroids. Microscope images show A) Spheroids before treatment and B) Spheroids after 72 h without treatment. C) Anticancer efficacy was evaluated using a U87 spheroid model with PTX as the model drug. The diameters were calculated as the mean of spheroid sizes from microscope images (*n* = 5). U87 multicellular cancer cell spheroids were incubated for 72 h with PTX at concentrations of 1 and 5 µg mL^−1^. Scale bars = 100 µm.

To evaluate the ability of the formulation to inhibit tumor growth, we exposed spheroids to the formulations loaded with PTX. Morphology and size change of spheroids were monitored for each formulation as shown in **Figure** **7**. In the absence of treatment, the spheroid size increased from 278 to 590 µm after 72 h of culture in full medium (Figure 7A). The addition of the different treatments based on PTX inhibited significantly the growth of the spheroids (Figure 7C). However, the volumes of the 3D spheroids exhibited no discernible differences following the treatments with free PTX and PTX‐loaded formulations, even at the highest concentrations tested (5µg mL^−1^). These observations confirmed that PTX‐loaded polymers and free PTX stopped the growth of spheroids to ≈300 µm after 72 h of treatment (Figure 7B).

The morphology of spheroids treated with free PTX indicated that the drug accumulated primarily at the periphery, leading to cell detachment from the spheroid surface while the core 3D structure remained largely unaffected. As the concentration of free PTX increased, more dead cells detached from the spheroids. In contrast, for PTX‐loaded BB polymers and polymeric micelle formulations, changes in spheroid morphology were likely due to the deeper penetration of BB polymers into the spheroid core, facilitated by passive internalization and diffusion pathways. This aligns with findings from other researchers, who have shown that particle morphology significantly influences cellular uptake. As the polymer formulation traveled through the 3D structure, PTX was gradually released within the spheroids,^[^ ^11^, ^58^ ^]^ ultimately disrupting spheroid integrity by killing U87 cells from within. These results not only clarify the cytotoxic mechanism of PTX‐loaded BB polymers compared to free PTX but also highlight the importance of PTX distribution in enhancing the efficacy of 3D tumor destruction.^[^ ^59^ ^]^

### 2.5 Biodistribution in Zebrafish Larvae

The zebrafish larvae model was used to assess the in vivo behavior and organ biodistribution of fluorescently labeled polymers after their injection into the bloodstream. The zebrafish model has recently gained popularity for screening nanomedicines due to several advantages, including tissue transparency that allows for high‐precision imaging, the presence of all major organs, and genetic similarities with mammals.^[^ ^60^, ^61^ ^]^

**Figure 8:**
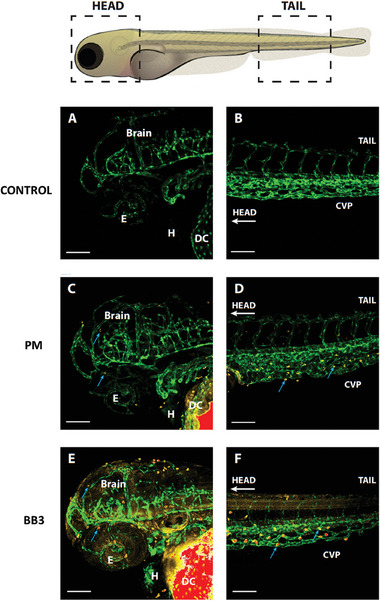
Head and caudal venous plexus confocal images of 50 hpf (flk1:EGFP) transgenic zebrafish larvae. After injection with Cy5 labeled BB3 or polymeric micelles, the images were acquired at 2 hh post injection. Images represented here are maximum projection images of z‐stack acquired as described in Material and methods. (A) and (B) control larvae (no injection); (C) and (D): larvae injected with polymeric micelles; E) and F): larvae injected with BB3 polymer particles. Yellow channel: Cy5‐labeled micelles or Cy5‐labeled BB polymer particles; Green channel: EGFP expressed in larvae vascular endothelial cells cytoplasm. Blue arrows indicate brain or caudal venous plexus macrophages. Abbreviations: E: Eyes; H: heart; DC: Duct of Cuvier; CVP: Caudal venous plexus. All images were acquired and processed using the same parameters in Zen 3.3, blue edition (Carl Zeiss GmbH). Scale bar: 100 µm.

The superiority of BB polymers over polymeric micelles in terms of tissue distribution and blood vessel evasion was confirmed after injecting Cy5‐labeled polymeric micelles and BB3 polymer into 50 hpf transgenic zebrafish larvae. A diffuse extravascular fluorescent signal was observed for BB3 polymer particles in the tail tissues (**Figure** **8**F) and in certain regions of the brain (Figure 8F), a signal that was not detected with linear polymer particles (Figure 8C,D). Additionally, a pronounced accumulation of the Cy5 signal was observed in the larvae's macrophages, located in both the caudal venous plexus and the head (blue arrows in Figure 8E,F) for BB3 polymer particles. A similar, but much less intense, signal was observed for polymeric micelles (blue arrows, Figure 8C,D). Some non‐specific labeling was noted near the injection site, specifically at the Duct of Cuvier (Figure 8C,E). Similarly, we also acquired images represented in 3D reconstructed images with Imaris of z‐stack in Figure S15 (Supporting Information).

In a previous study on BB polymers made with oligo PEG methacrylate in the side chains, it was observed that the diffusivity of BB polymer in larvae tissues was superior to that of PEGylated solid particles.^[^ ^56^ ^]^ Imaging evidenced that BB polymers chains were able to freely leave the vascular compartment, whereas PEGylated solid particles of similar hydrodynamic size remained in circulation for an extended period of time. In contrast, the present study reveals, along with an extra‐vascular diffusion, the accumulation of BB polymer fluorescence in larval macrophages which was not detected in the previous study. It can be hypothesized that this difference in biodistribution behavior is attributable to the distinctive core–shell structure of the BB polymer.

However, the chemistry of the outer layer of the particle may also play a significant role. In a separate study, we previously demonstrated that solid particles composed of P(D,L)LA‐b‐PMPC diblock polymer, when injected into the bloodstream of zebrafish larvae, exhibited a short circulation time and accumulated at the vessel walls, particularly in the caudal venous plexus.^[^ ^62^ ^]^ This widespread accumulation in the tortuous part of the caudal venous plexus was likely due to multiple factors, including nanomaterial adhesion to the surface of endothelial cells and uptake by endothelial cells or macrophages.

The PMPC outer layers seem to increase the tropism for macrophages in both polymeric micelles and BB polymer particles. This is counterintuitive, as PMPC surfaces are known for their antifouling properties, which typically contribute to prolonged blood circulation in vivo.

These earlier findings align with the present data, confirming the impact of polymer architecture on rapid blood compartment evasion for BB polymer particles compared to micelles or solid polymeric nanoparticles. They also highlight the role of the PMPC outer layer in influencing biodistribution. The current results provide valuable insights into the ability of nanocarriers to persist in circulation, interact with the vascular endothelium, diffuse into tissue, and be cleared from the bloodstream. Taken together, these findings confirm and expand upon previous observations of the unique properties of BB polymers, which were also evident in 2D cell culture and spheroid penetration assays conducted in this study.

## 3 Conclusion

In a nutshell, a library of cylindrical brush structure‐based core–shell BB polymers and micelles with spherical structures were prepared and used as nanocarriers for RhB and PTX. These unimolecular brushes showed a high loading capacity of molecules compared to conventional micelles based on linear di‐block polymers. Furthermore, the worm‐like shape of BB polymer with a low number of sidechains exhibited improved performances in cellular uptake and penetration into 2D and 3D cell models. Based on the anticancer efficacy results, this core–shell structure of BB polymers could be promising for the construction of nanocarriers with high drug loading capacity and deeper penetration into the targeted sites.

## 4 Experimental Section

### Materials

The polymeric backbone poly(HEMA‐TMS)‐b‐(MMA) was synthesized following the prior method (Supporting Information).^[^ ^16^ ^]^ Methyl methacrylate (MMA, 99%, Aldrich) and 2‐(trimethylsilyloxy)ethyl methacrylate (HEMA‐TMS, Aldrich) were passed through a column filled with basic alumina prior to use. 2‐Methacryloyloxyethyl phosphorylcholine (MPC, 97%, Aldrich) was recrystallized from acetonitrile and dried under vacuum overnight at room temperature before polymerization. Copper(I) bromide (Cu^I^ Br, 99.999%, Aldrich), copper(II) bromide (Cu^II^Br_2_, 99.999%, Aldrich), copper(I) chloride (Cu^I^ Cl, ≥99.995% trace metals basis, Aldrich), copper(II) chloride (Cu^II^Cl_2_, ≥99.995% trace metals basis, anhydrous, Aldrich), 2,2′‐bipyridyl (bpy, 99%, Aldrich), 4,4′‐ Dinonyl‐2,2′‐dipyridyl (dNbpy, 97%, Aldrich), potassium fluoride (KF, 99%, spray‐dried, Aldrich), tetrabutylammonium fluoride (TBAF, 1M solution in THF, Aldrich), α‐bromoisobutyryl bromide (98%, Aldrich), bromoethane (98%, Aldrich), and tributyltin hydride (97%, Aldrich) were used without any additional purification. Ethylene bis(2‐bromoisobutyrate) (diBr) was synthesized according to previously published procedures. D,L‐Lactide was recrystallized from ethanol and toluene and then dried under vacuum with P_2_O_5_ before reaction. 1,8‐Diazabicyclo(5.4.0) under‐7‐ene (DBU) was distilled under vacuum by heating the flask in an oil bath (vacuum: 11 mbar; oil bath: 147 °C). All other solvents were used as received.

### Synthesis of HEMA‐MMA with Different Units of OH (1)

The polymeric backbone (0.35g; 0.00271 mmol polymer, 0.529 mmol HEMA‐TMS unit) was dried and redissolved in 20 mL THF. Potassium fluoride (0.061 g, 1.05 mmol) and 0.79 mL (0.79 mmol) of tetra‐n‐butylammonium fluoride (TBAF) were added. This mixture was stirred for 24 h. THF was partly evaporated, and the mixture was then precipitated in 100 mL distilled water containing two drops of HCl. The polymer was re‐dissolved in THF and dialysis against THF. After evaporation of the solvent, the polymer was dried and analyzed with GPC. The NMR spectrum was recorded by using DMSO‐d6 as solvent. About 180 mg polymer was obtained (yield 51.3%).

### Ring‐Opening Polymerization of P(D,L)LA and End‐Groups Functionalization

The deprotected polymer (22.7 mg, 0.228 µmol; 0.094 mmol HEMA group) and (D,L)‐dilactide (595 mg, 4.14 mmol) were placed in a dried flask, evacuated, and refilled with argon three times. Dry DMF (4.5 mL) was then added under nitrogen, and the mixture was stirred until all polymer dissolved. DBU (6.3 µL) was then injected to initiate the polymerization, and the reaction was carried out at room temperature for 1.5 h. The polymerization was quenched by adding benzoic acid (50 mg). The polymer solution was diluted with THF and dialysis against THF using tubes with a pore size molar mass cut off 50 000 kDa for 24h. The polymer solution was concentrated and precipitated into cold methanol. The polymer was dried under vacuum and then dried with P_2_O_5_.

Afterward, 100 mg (0.018 mmol of OH groups) of (PMMA‐b‐PHEMA)‐g‐P(D,L)LA was dissolved in 5 mL of DCM and 6.4 µL (46.2 umol) of TEA was added at 0 °C. Next, 5.7 µL (46.1 µmol) of 2‐bromoisobutyryl bromide in 2 mL of DCM was added dropwise into the prepared mixture. After at least 30 min, the reaction mixture temperature was raised to room temperature and kept under stirring for 24 h. The solids were filtered off, and the solution was concentrated and precipitated into cold methanol. The precipitated macroinitiator was re‐dissolved in chloroform and passed through a short column filled with basic alumina. The filtrate was re‐precipitated three times from chloroform into hexanes and dried under vacuum overnight at room temperature.

### Synthesis of PMPC by ATRP

A dry 5 mL Schlenk flask was charged with the macroinitiator (10.2 mg, 2.8 µmol of BiBEM), 2‐methacryloyloxyethyl phosphorylcholine (2.5 g, 8.5 mmol), 2,2′‐bipyridyl (15.0 mg, 0.0960 µmol), Cu^II^Cl_2_ (as a stock solution, 0.76 mg, 0.056 mmol), acetonitrile (3.0 mL) and methanol (7.0 mL). The solution was degassed by three freeze‐pump‐thaw cycles. After the final cycle, Cu^I^Cl (4.2 mg, 0.042 µmol) was added followed by thawing the reaction mixture under a nitrogen atmosphere, and the flask was immersed in an oil bath thermostated at 50 °C. The reaction was stopped by exposing the solution to air. The (PMMA‐b‐PHEMA)‐g‐(P(D,L)LA‐PMPC) polymer was purified by dialysis against MeOH for 48 h using tubes with a pore size molar mass cut off 50 kDa.

### Polymer Characterizations

Proton nuclear magnetic resonance (^1^ H NMR) spectroscopy was performed using a Bruker 600 MHz spectrometer. Deuterated chloroform (CDCl_3_), Deuterated DMSO (DMSO‐d_6_), and deuterated methanol (CD_3_OD) were used solvents.

Apparent molecular weights and molecular weight distribution measurements of polymeric backbones and BB grafted PLA were measured by gel permeation chromatography (GPC) using Polymer Standards Services (PSS) columns, with THF as eluent at a constant flow rate of 0.5 mL min^−1^ at 25 °C and differential refractive index (RI) detector (Waters and Wyatt). The apparent number‐average molecular weights (*M_n_*) and molecular weight distribution (*M_w_/M_n_*) were determined with a calibration based on dn/dc as the standards.

The core–shell BB polymers were imaged by atomic force microscopy (AFM) (ICON FastScan, Bruker). The purified polymers were diluted with Milli‐Q water at different concentrations varied from 100 to 10µg mL^−1^ and deposited on a freshly cleaved mica surface. The BB polymers were left for a few seconds to adsorb, and the supernatant was then rinsed three times. The surface was nitrogen‐dried prior to AFM measurements. The AFM equipped with nanoscope VIII controller (Digital Instruments) was set on the peak force QNM mode.

The drug loading and release were quantified by a high‐performance liquid chromatography (HPLC) system (Shimadzu Prominence – Shimadzu USA Manufacturing Inc.) composed of a pump (LC‐20A HT), a UV–vis detector (SPD‐20A), a column oven (CTO‐20A), a syringe loading sample injector (SIL‐20A), and a Hypersil GOLD PFP column (150 mm × 4.6 mm i.d., 5 µm particle size). For the HPLC analysis of Rhodamine B, the mobile phase was Milli‐Q water–methanol (25:75 v/v) at a flow rate of 0.8 mL min^−1^, with 20 µL injection volume, and the column oven temperature was set at 30 °C. For Paclitaxel, the mobile phase was acetonitrile–water (adjusted to pH 5 with KH_2_PO_4_) (40:60 v/v) at a flow rate of 1.0 mL min^−1^ and 20 µL injection volume.

### Payloads Encapsulation and In Vitro Drug Release

The solvent exchange method was used for preparing drug‐loaded BB polymers and PM. Briefly, rhodamine B and paclitaxel, were separately encapsulated into polymeric structures. For the individual loading experiment, the drug molecule was dissolved into DMF at a concentration of 200 µg mL^−1^. The polymer solution in methanol was prepared to a final concentration of 600 µg/mL. The molecule and polymer solutions were mixed and dialyzed against MilliQ water overnight using a dialysis membrane (MWCO: 12 000 Da) to remove DMF, methanol, and unloaded molecules.

The dialyzed suspension was centrifuged, and the supernatant was measured by HPLC to calculate the amount of free drug. The below formulas were applied to determine the drug loading efficiency (DLE) and drug loading capacity (DLC):

(1) $$DLE\;\%\;=\frac{\mathrm{weight}\;\mathrm{of}\;\mathrm{loaded}\;\mathrm{drug}\;\mathrm{in}\;\mathrm{BBs}}{\mathrm{weight}\;\mathrm{of}\;\mathrm{drug}\;\mathrm{in}\;\mathrm{feed}}\;X\;100$$

(2) $$DLC\;\%\;=\frac{\mathrm{weight}\;\mathrm{of}\;\mathrm{loaded}\;\mathrm{drug}\;\mathrm{in}\;\mathrm{BBs}}{\mathrm{total}\;\mathrm{weight}\;\mathrm{of}\;\mathrm{BBs}}\;X\;100$$

PBS solutions at pH 7.4 were used at 37 °C for the investigation of in vitro drug release profiles of drug‐loaded BB polymer‐carriers. 3 mL of the drug‐loaded BB polymer solution was put in a dialysis bag (MWCO: 12 000 Da) and stirred in 20 mL of PBS solution at 37 °C. Afterward, 3 mL of the solution was taken out at different time‐intervals (1, 2, 6, 12, 24, and 48 h) to quantify the concentration of released molecules by measuring the absorbance at 550 nm for Rhodamine B and 230 nm for Paclitaxel by HPLC. 3 mL of the buffer solution was added at the same condition to maintain the volume of release media.

### 2D Cell Culture

Caco‐2 (Human Colorectal Adenocarcinoma), HepG2 (Human hepatoma), and U87 (Human glioblastoma) cells were grown in DMEM medium, supplemented with 10% FBS and 1% Penicillin/Streptomycin mix (Wisent, Canada) and kept at 37 °C, 5% CO2 in a humidified atmosphere. Cells were grown in T75 flasks (Sarstedt, Montreal, Canada). After detaching with 0.25% Trypsin/EDTA solution (Wisent, Canada), the number of cells was counted using a manual hemocytometer.

### Cytotoxicity Assays

The cytotoxicity of BB and linear polymers was determined on Caco‐2, U87, and HepG2 cells. About 100 µL of cell suspension in complete culture medium at a concentration of 1.5 × 10^5^ cells mL^−1^ was added to each well of 96‐well plates (Sarstedt, Canada). After 24 h, the cell medium was removed and replaced with 90 µL of fresh complete medium. BBs suspensions in PBS were added at final concentrations varying from 1000 to 0.5 µg mL^−1^ (*n* = 5 for each polymer and concentration). Plates were incubated at 37 °C in a humidified 5% CO_2_ atmosphere for 24 h or 72 h. After media removal, 10 µL of MTS solution (5 mg mL^−1^) (Abcam, Canada) was added to 90 µL of complete media in each well. After 4 h of cell incubation at 37oC, the absorbance was read at 490 nm on a plate reader (Spark, Tecan, Austria). Cell proliferation calculations used untreated cells as 100% cell proliferation control and complete medium as background absorbance.

### Cytotoxicity of Paclitaxel‐Loaded Formulations (MTS Assay and Imaging)

Similarly, cytotoxicity of PTX and PTX loaded BB and linear diblock polymers was assessed on Caco‐2 and U87 cells. For free PTX assessment, a PTX stock solution, 1 mg mL^−1^ in DMF was serially diluted in PBS to get solution concentrations ranging from 200 µg mL^−1^ to 20 ng mL^−1^ (final concentration in cell culture medium). Cytotoxicity of PTX‐loaded nanoformulation on Caco2 and U87 cells was tested in the range of 0.5–10 µg mL^−1^ of PTX. After nano formulation preparation, PTX was quantified by HPLC and diluted in a cell culture medium. Afterward, Caco2 and U87 cells were exposed to PTX solutions for 72 h before the cell culture medium was changed with 90 µL of fresh medium and 10 µL MTS (Abcam, Canada) added in each well. After a 4 h incubation at 37 °C, plates were read using a Microplate reader (Spark Cyto, Tecan Trading AG, Switzerland), and the effect on cell proliferation was calculated using the following formula:

(3) $$Cell\;proliferation\;\left(\%\right)=\left(\frac{\mathrm{Number}\;\mathrm{of}\;\mathrm{live}\;\mathrm{cells}}{\mathrm{Toal}\;\mathrm{number}\;\mathrm{of}\;\mathrm{cells}\;}\right)\times 100$$

Besides that, before MTS addition, to investigate the morphology change of cells after the PTX treatments, the Caco‐2/U87 cell monolayers were imaged in phase contrast on a Zeiss Axio Observer inverted microscope (Zeiss, Germany) using a 4X magnification for a large field of view. The image was recorded with a AxioCam MRm camera and treated in Zen blue software (Carl Zeiss, Germany).

### In Vitro Cellular Uptake—Flow Cytometry

Caco‐2/U87 cells were seeded in 6‐well plates (Sartedt, Canada) at a concentration of 10^5^ cells mL^−1^ in 2 mL of complete medium. When the cells reached 90% confluence, the medium was changed, and formulations were added at a concentration of 3–15 µg mL^−1^ (based on RhB concentration in the BB and linear polymers formulations).

After the incubation period, the medium was removed, and the cell monolayer was washed twice with cold sterile PBS. Then, 200 µL of 0.25% Trypsin/EDTA (Wisent, Qc Canada) was added, and the plates were incubated at 37 °C for 15 min. Detached cells were transferred to RIA tubes and washed (centrifuged and resuspended) twice with cold PBS before being fixed in 0.5 mL of 0.5% PFA/PBS buffer. RhB uptake into Caco2/U87 cells was quantified using flow cytometry on a Cytoflex cytometer (Beckman Coulter). Around 20 000 events were recorded for each condition. Data were treated based on the mean of the intensities with *n* = 5.

### In Vitro Cellular Uptake—Confocal Images

Caco‐2/U87 cells were seeded on sterilized glass disks in 24‐well plate at a concentration of 5 × 10^4^ cells/wells. After a 48 h attachment and growth period, the culture medium was replaced with fresh medium, and RhB‐loaded nanoformulations (BB and linear polymers) were added at a concentration of 3–15 µg mL^−1^ (based on RhB concentration). Free RhB was used as a control using the same concentrations.

Cells were incubated at 37 °C in 5% CO_2_ atmosphere. At the end of the incubation time, the medium was removed, and cell monolayers were washed with ice‐cold PBS twice. Caco‐2/U87 cell nuclei were stained with Hoechst 33342 Trihydrochloride, Trihydrate solution in PBS (Thermo‐Fisher, Canada) after cells being fixated with 4% (w/v) Paraformaldehyde (PFA) in PBS pH 7.4. After extensive washing with PBS to remove unbound dye and PFA, glass slides were mounted using a Prolong antifade mounting medium (ThermoFisher, Canada) and cells were imaged in fluorescence (Red and Blue channels to detect respectively, RhB uptake and nucleus staining) on a confocal microscope (Zeiss LSM 880, Airyscan, inverted, Germany).

### 3D Tumor Spheroid Penetration

The 3D tumor spheroids were prepared using ultralow adhesion round bottom plates (Sarstedt, Canada). U87 cells were seeded in low attachment 96‐well plates with a cell concentration of 2 × 10^3^ cells/well in 200 µL complete medium. After a short centrifugation, the cells were incubated for 3 days until the cell spheroids formed and the size was monitored in phase contrast image on Zeiss Axio Obsever microscope (at X4 magnification). After replacing with fresh medium, the spheroids were incubated with RhB or RhB‐loaded BB and linear polymers (concentration of RhB in the range of 3–10 µg mL^−1^). After 4 h incubation, the spheroids were washed with PBS twice and fixed with 4% (w/v) Paraformaldehyde (PFA) in PBS followed by a cell nucleus staining with Hoechst 33 342. After extensive washing with PBS to remove the unbound dye and PFA, the spheroids were transferred to a glass cover box and the confocal laser scanning microscope (Zeiss LSM 880, Airyscan, inverted, Germany) was used to investigate the distribution of fluorescence.

### Cytotoxicity Test on 3D Tumor Spheroid

U87 cell‐based 3D spheroids were prepared according to the previously described protocol above. Following the initial growth period, they were incubated with PTX and PTX‐loaded BB and linear diblock polymers (concentration of PTX in the range of 3–10 µg mL^−1^). After 72 h of incubation, the morphology and size of the U87 spheroids were observed under phase contrast on a Zeiss Axio Observer inverted microscope (Zeiss, Germany) at 4X magnification for a large field of view (*n* = 5 for each polymer and concentration). Images were captured with an AxioCam MRm camera and processed in Zen Blue software (Carl Zeiss, Germany).

### Injections in Zebrafish Larvae

Transgenic (flk1:EGFP) zebrafish (Danio rerio) larvae eggs (GFP‐labeled fluorescent endothelial cells) were obtained from the Institute National de la Recherche Scientific Armand‐Frappier Santé Biotechnologie research center.^[^ ^63^ ^]^ The experimental procedures were conducted in concordance with the Canadian Council on Animal Care (CCAC) guidelines. The eggs were incubated for 24 h in E3 medium at 28 °C after which the eggs were transferred into E3 medium containing propylthiouracil (PTU) with a concentration of 0.003% w/v. At 50 hpf, the larvae were anesthetized by incubating them in 0.2mg mL^−1^ tricaine (Sigma‐Aldrich) after which they were transferred into a Petri dish and immobilized with 1% w/v low melting point agarose (Fisher Bioreagents).

Cy5 dye as a model near‐infrared fluorescent probe was loaded into the BB3 and PM using the dialysis method as described in Section 2.6. Afterward, the loaded Cy5 was quantified by UV–vis spectroscopy (Malvern, UK) at 640 nm. The concentration of Cy5 was identical for all the formulations and fixed at 50 µg mL^−1^. Fluorescent formulation suspension (4nL) was injected into the duct of the curvier of the larvae using an Eppendorf Femtojet 4i injector as previously described.^[^ ^56^, ^62^ ^]^ The sub‐caudal region and the head of the larvae were imaged at 2 h post‐injection with a Zeiss LSM 700 confocal microscope (Carl Zeiss, Germany), using a 10 X objective. Separate channels were set up for Cy5 (polymer labeling) and GFP. Imaging parameters lasers were kept identical for all larvae. Images were treated using Zen blue software (Carl Zeiss, Germany) and finally, 3D images were constructed from z‐stacks with Oxford IMARIS program version 9.2.1 (Oxford Instruments, Bitplane Inc. Concord, MA USA) at IRIC (Université de Montréal).

### Statistical Analysis

Numerical results are presented as mean ± SD. The comparison between different groups was analyzed via the student's t‐test using the Analysis mode of Sigmaplot (version 7.0), and “p” less than 0.05 was regarded as statistically significant.

## Conflict of Interest

The authors declare no conflict of interest.

## Supporting information



Supporting Information

## Data Availability

The data that support the findings of this study are available from the corresponding author upon reasonable request.
