# Supplementary material for: Core–Shell Bottlebrush Polymers: Unmatched Delivery of Small Active Compounds Deep Into Tissues
Source: Small. 2024 Dec 16;21(5):2408616. doi: 10.1002/smll.202408616 (PMC11798360; doi:10.1002/smll.202408616)
Supplement: Supplementary file 1 — Supporting Information [file SMLL-21-2408616-s001.docx]

Supporting Information

Core-Shell Bottlebrush Polymers: Unmatched Delivery of Small Active Compounds Deep into Tissues.

Quoc Thang Phan ^a^, Jean-Michel Rabanel ^a, b^, Dikran Mekhjian ^a^, Justine Saber ^a^, Araceli Garcia Ac ^a^, Hu Zhang ^a^, Victor Passos Gibson ^c^, Charlotte Zaouter ^d^, Pierre Hardy ^c^, Shunmoogum A. Patten ^d^, Daria Boffito ^e^, Xavier Banquy ^a, f, g, *^

^a^ Faculty of Pharmacy, Université de Montréal, 2940 Chemin de Polytechnique, Montréal, Québec H3T 1J4, Canada

^b^ School of Pharmaceutical Sciences, Faculty of Medicine, University of Ottawa, Roger Guindon Hall, 451 Smyth Rd, Ottawa, ON K1H 8M5 Canada

^c^ Department of Pharmacology and Physiology, Université de Montréal, Montréal, QC H3T 1J4, Canada

^d^ INRS Centre Armand-Frappier Santé Biotechnologie, 531, boul. des Prairies, Laval, QC, Canada H7V 1B7

^e^ Department of Chemical Engineering, Polytechnique Montréal, 2500 Chemin de Polytechnique, Montréal, Québec H3C 3A7, Canada

^f^ Biomedical Engineering Institute, Université de Montréal, 2940 Chemin de Polytechnique, Montréal, Québec H3T 1J4, Canada

^g^ Chemistry Department, Faculty of Arts and Sciences, Université de Montréal, 2940 Chemin de Polytechnique, Montréal, Québec H3T 1J4, Canada***

** Corresponding Author: Xavier Banquy; E-mail address: xavier.banquy@umontreal.ca*

- 1. Supplementary method:

Nano-flow cytometry (NanoFCM)

Nanoanalyzer (NanoFCM, China) is used to characterize the Cy5 encapsulation within the BB and linear polymers.

Dye-loaded nanoparticles were prepared as described in Section 2.6 with BB3/PM polymers and Cy5 as fluorescent dye (polymer: dye (w/w) = 3:1). After overnight dialysis, Cy5-loaded nano formulations and empty nano formulations as a control were diluted to a particle concentration at ∼10^9^ particles/mL and loaded to the Nanoanalyzer system.

The analyses were carried out as described by previous study, a 642 nm laser was used as the excitation source, and the side scatter and intrinsic fluorescence of Cy5 emitted by single Cy5-NPs were detected by two avalanche photodiodes (APDs), respectively. And the measurement was performed with a number of events between 3,500 and 12,000 [1].

- 1. Supplementary figures:

|  | Backbone  Conversion ratio (%) * | Backbone  DP of polymer** | Mn | PLA  Conversion ratio (%) | PLA  DP of polymer | Mn | PMPC  Conversion ratio (%) | PMPC  DP of polymer | Mn |
| --- | --- | --- | --- | --- | --- | --- | --- | --- | --- |
| BB1 | 27.5 | 1100 | 189,310 | 14% | 14 | 1,009,822 | 35% | 140 | 35,161,708 |
| BB2 | 30 | 1200 | 180,120 | 17% | 17 | 934,920 | 37.5% | 150 | 38,555,026 |
| BB3 | 20 | 800 | 78,060 | 22% | 22 | 371,100 | 45% | 180 | 13,608,448 |
| PM | x | x | x | 21% | 21 | 1554 | 33.75% | 135 | 43,849 |

** Conversion ratio was calculated by NMR.*

*** DP of polymer calculated by multiply fed number (2000 for backbone; 100 for PLA; and 400 for PMPC) with conversion ratio.*

*and Mn after each polymerization step were calculated by NMR.*

Table S1: The compositions of BB and linear polymers including the conversion ratio and actual number of each polymer after 3 steps polymerization.

*
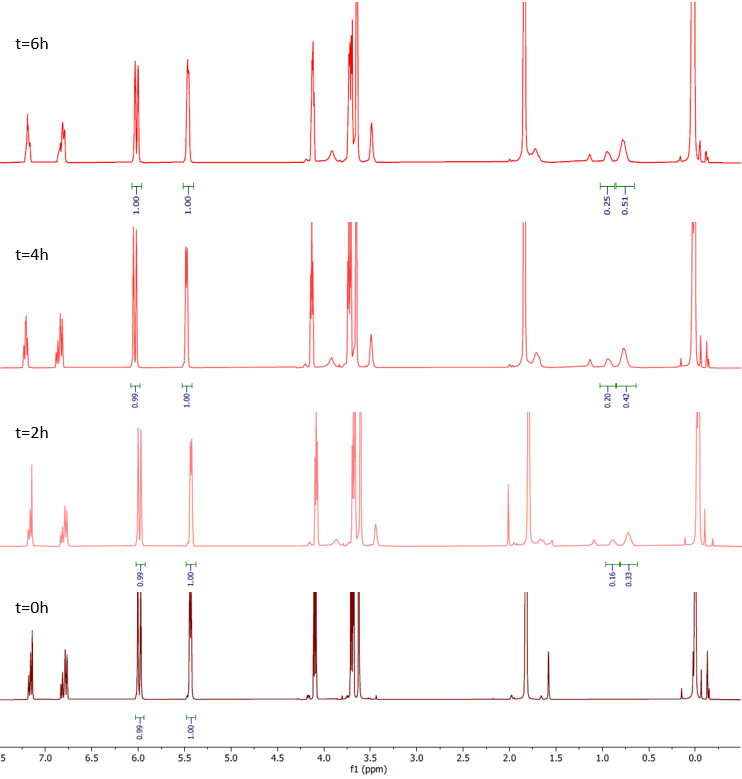
*

**Figure S1.**  **^1^H-NMR spectra of the conversion ratio of polymeric backbones polymerization**. The conversion ratio was calculated by comparing the integrals of -CH_3_ in polymer at 0.6 and 0.8 ppm with -CH_2_ of monomer at 5.5 and 6.0 ppm.


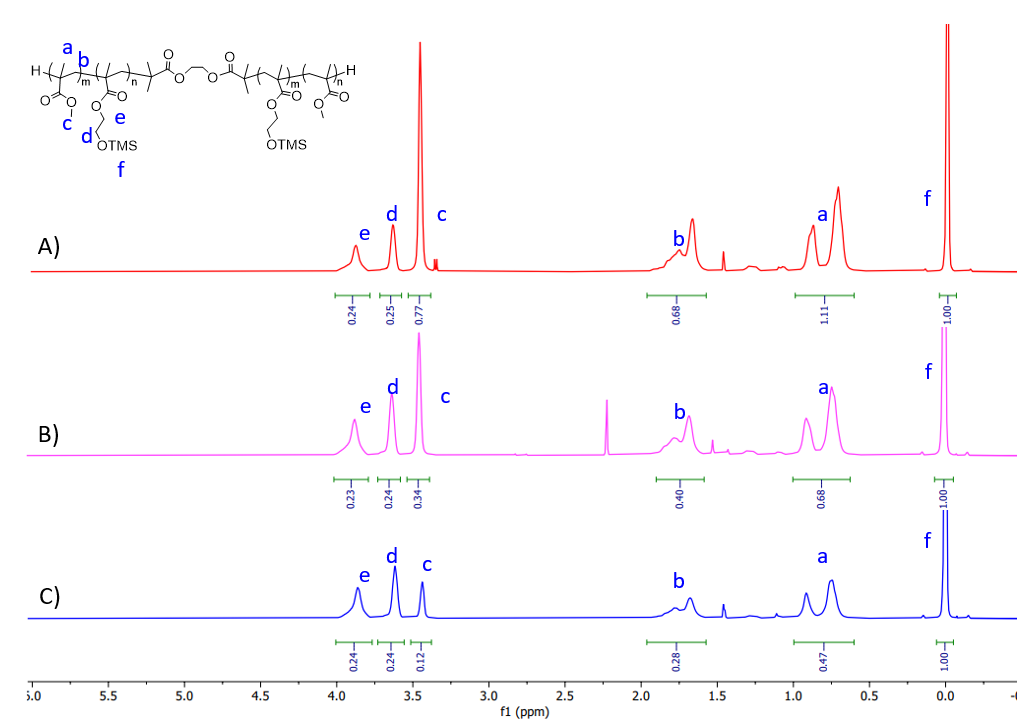


**Figure S2.**  **^1^H-NMR spectra of the polymeric backbones with different grafting density in CDCl_3_**. (A) G=0.3, (B) G=0.5, and (C) G=0.72 (grafting density was calculated by comparing the integral of “c” and “f”).


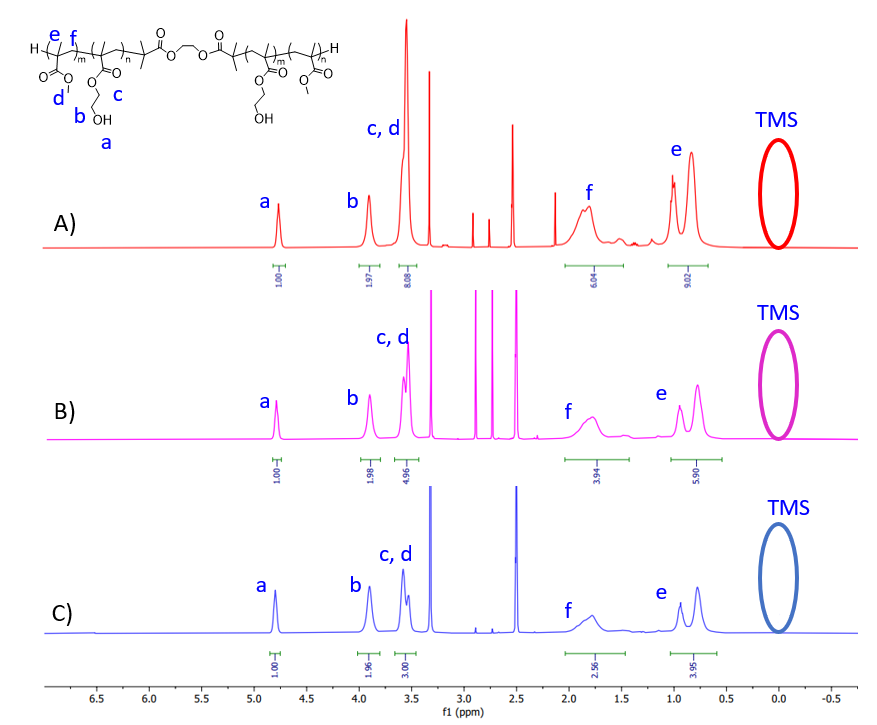


Fig S3. ^1^H-NMR spectra of the polymeric backbones after removal of TMS in (CD_3_)_2_SO. (A) G=0.3, (B) G=0.5, and (C) G=0.72 (peaks of TMS at 0.0 ppm disappeared and there was a new peak of -OH at 4.8 ppm)


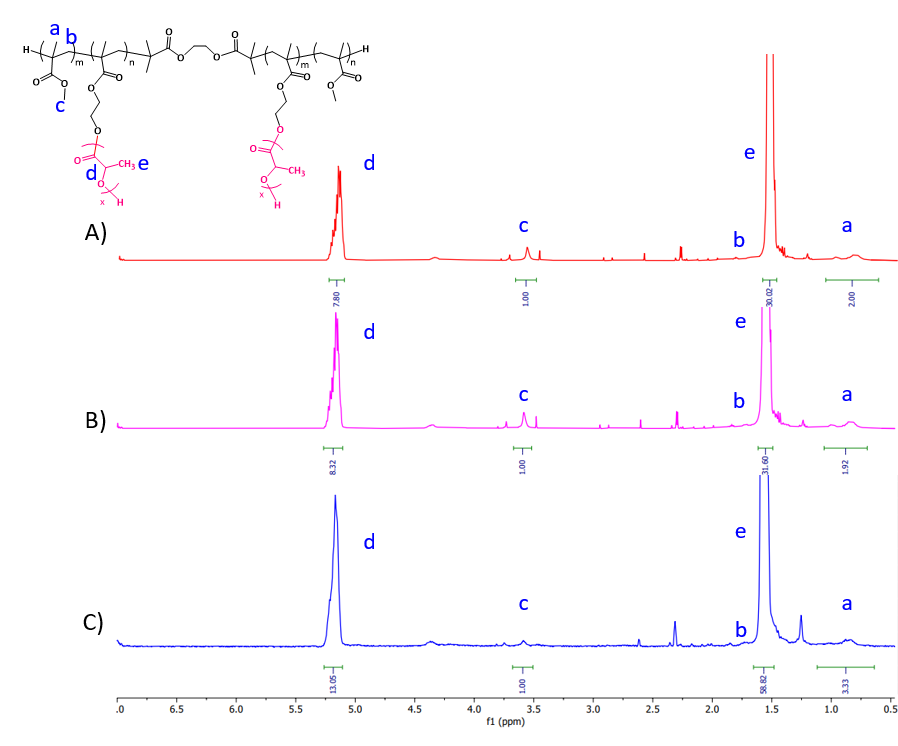


Fig S4. ^1^H-NMR spectra of the BB polymers after the grafting of P(D,L)LA in CDCl_3_. (A) G=0.3, (B) G=0.5, and (C) G=0.72 (DP of P(D,L)LA was calculated by comparing the integral of “c” and “d”).


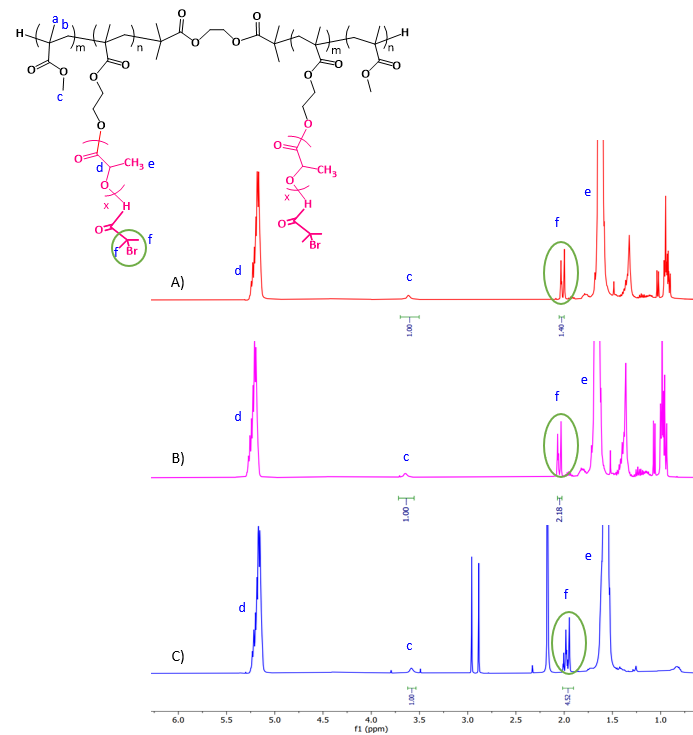


Fig S5. ^1^H-NMR spectra of P(D,L)LA grafted BB polymers after the end-group bromination in CDCl3. (A) G=0.3, (B) G=0.5, and (C) G=0.72 (The complete bromination was confirmed by comparing the integral of “c” and “f”).


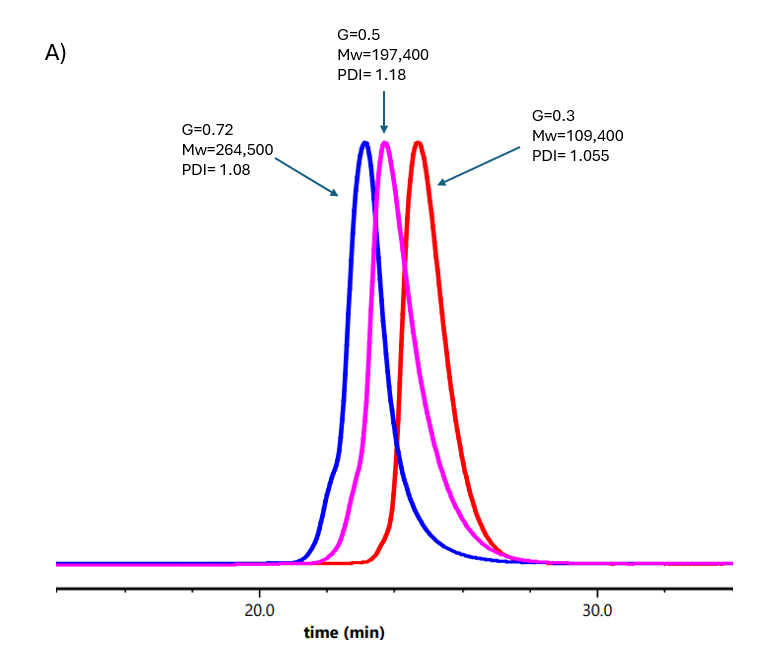


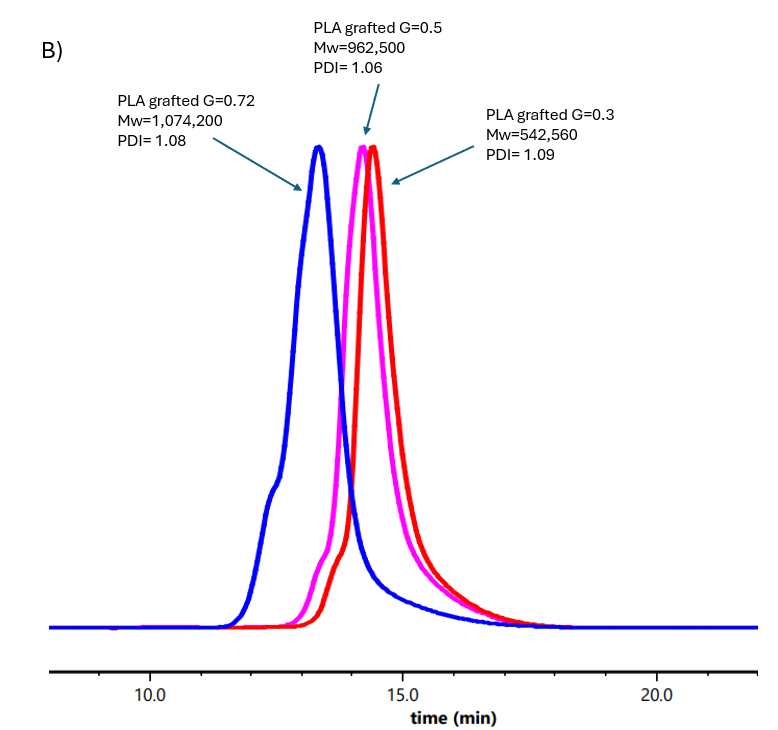


Fig. S6. GPC traces of the backbone (HEMA-TMS-co-MMA) and BB-P(D,L)LA polymer using THF as eluent. (A) polymeric backbones with different grafting density, and (B) P(D,L)LA grafted on the three backbones.


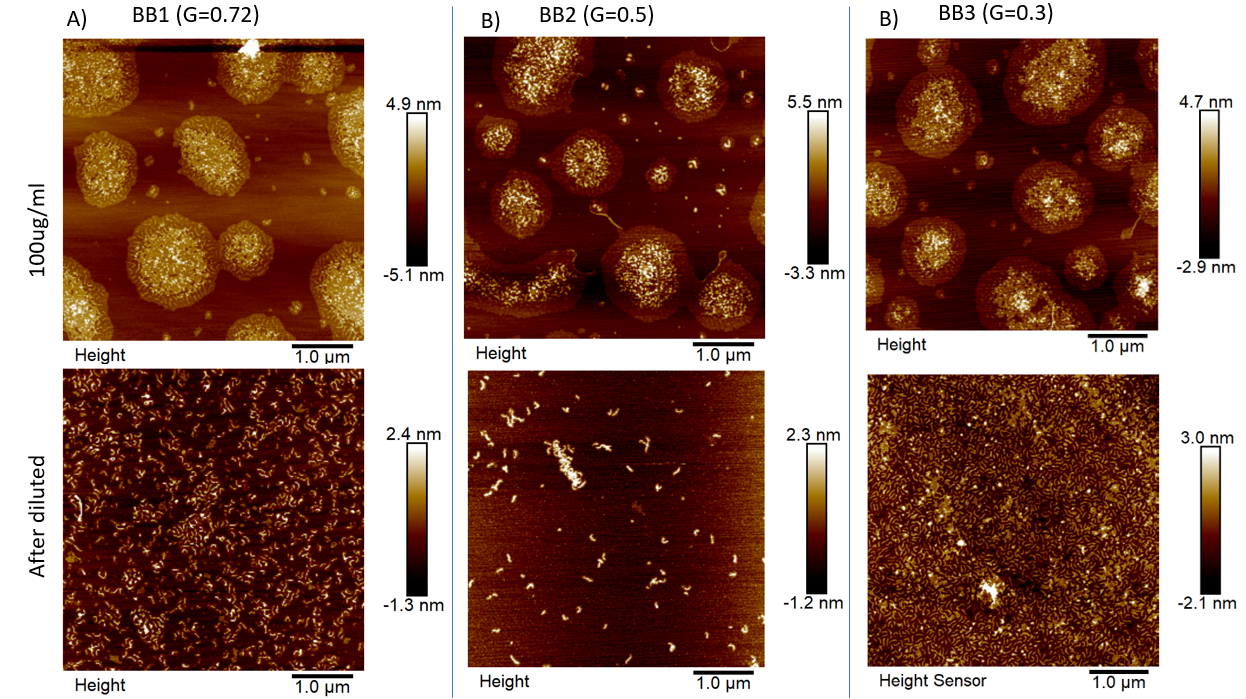


Fig. S7. Self-assembly properties of BB polymers depending on concentrations. The morphologies of polymers were evaluated by AFM images - A) BB1 (G=0.72); B) BB2 (G=0.5); and C) BB3 (G=0.3). (The upper panel are the morphologies of polymers at high concentration above 100µg/ml and lower panel are images after dilution to 10µg/ml).


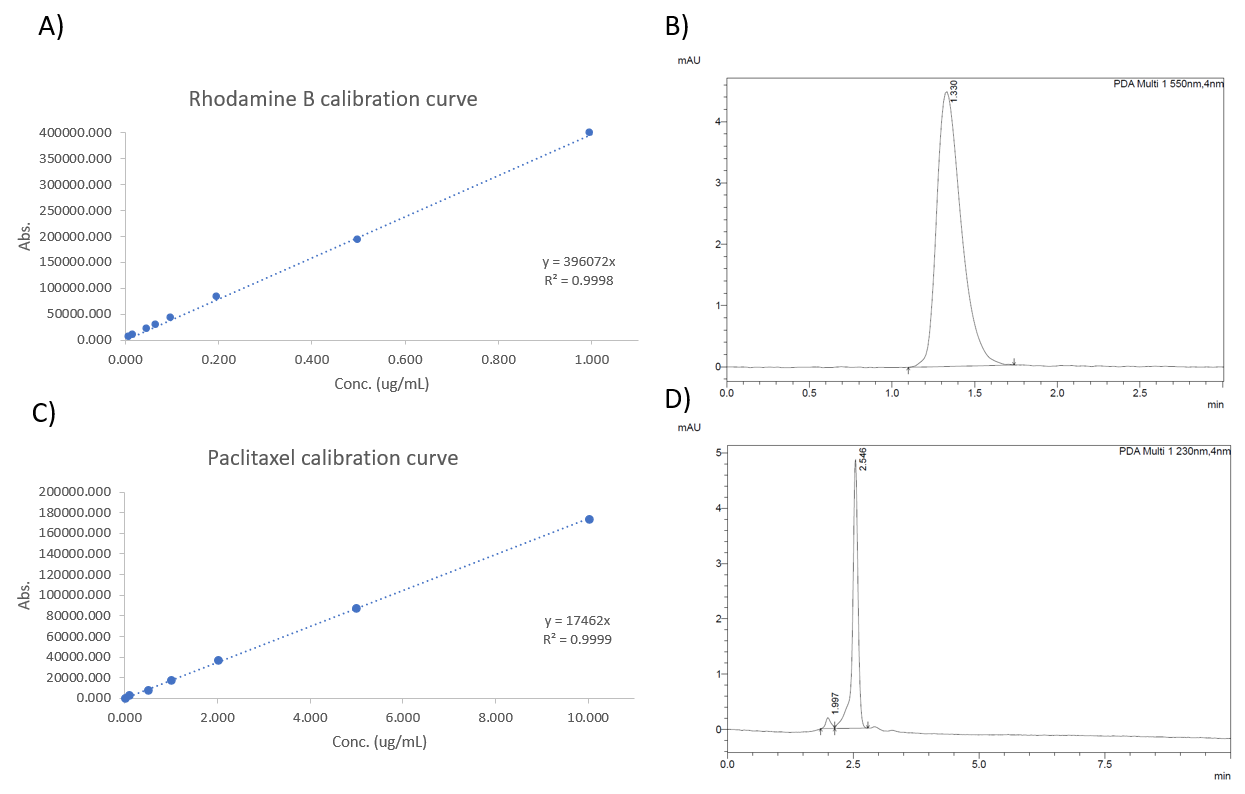


Fig. S8. HPLC quantification of RhB and PTX. A) Calibration curve of RhB; B) HPLC graph of RhB; C) Calibration curve of PTX and D) HPLC graph of PTX.


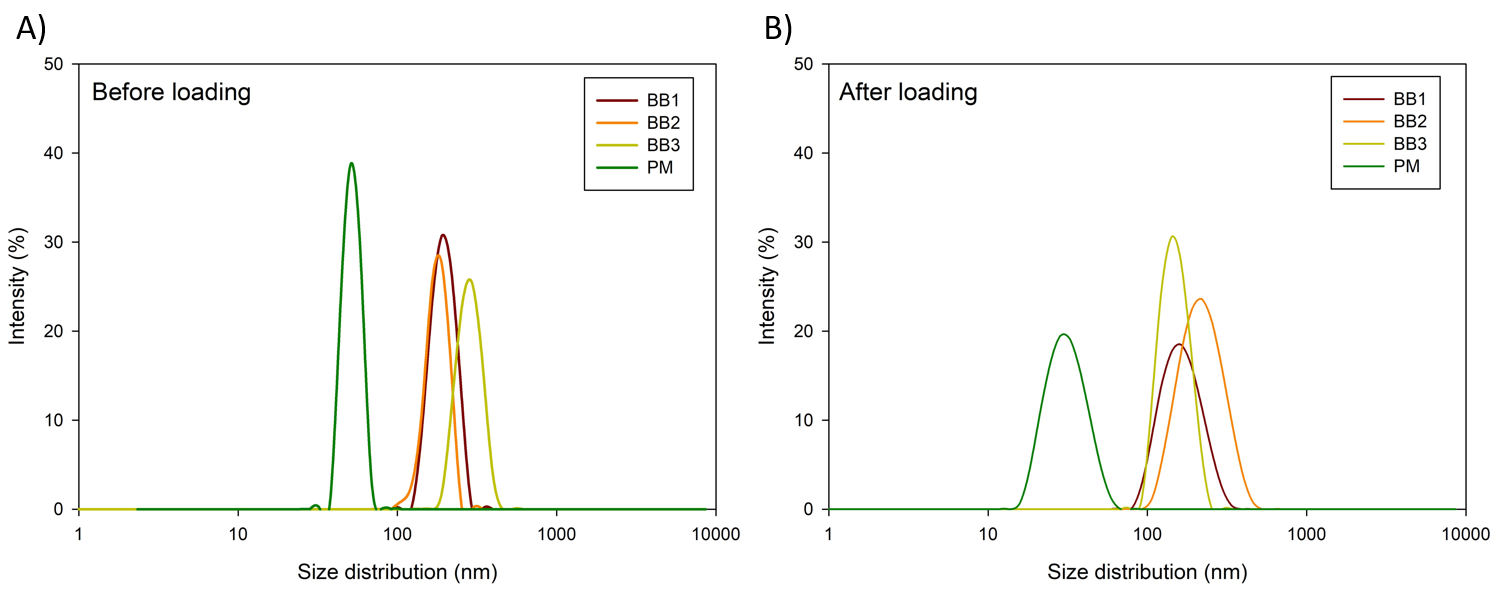


Fig. S9. Size distribution of BB polymers and PM before and after the loading of PTX. A) Before loading and B) After loading.


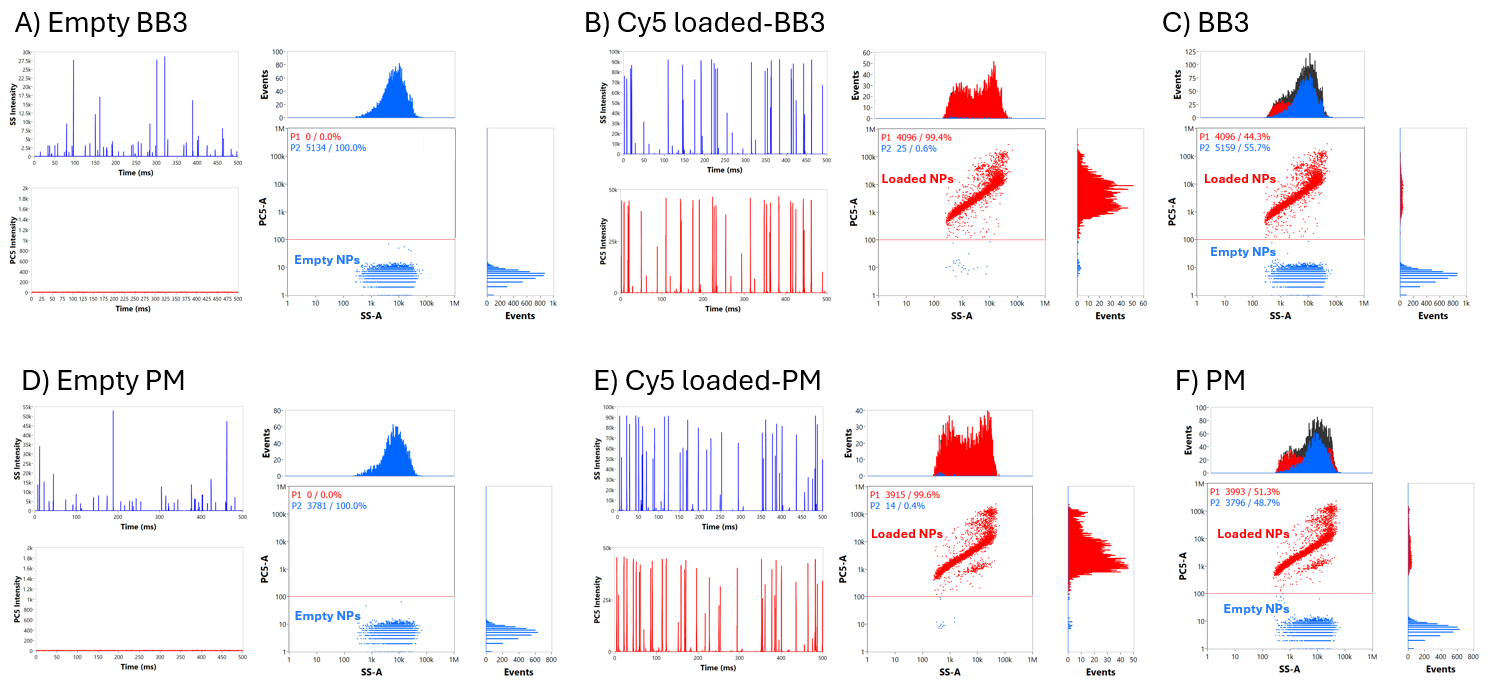


Fig. S10. Characterization of Cy5 loaded BB and linear polymers using Nanoflow cytometry. A, D) Representative side scattering (SS) and Cy5 fluorescence traces for empty nano formulations; B, E) for Cy5 loaded BB3 and PM; and C, F) Compiled scatter plots of fluorescence signal versus SS signal for empty and loaded nano formulations.


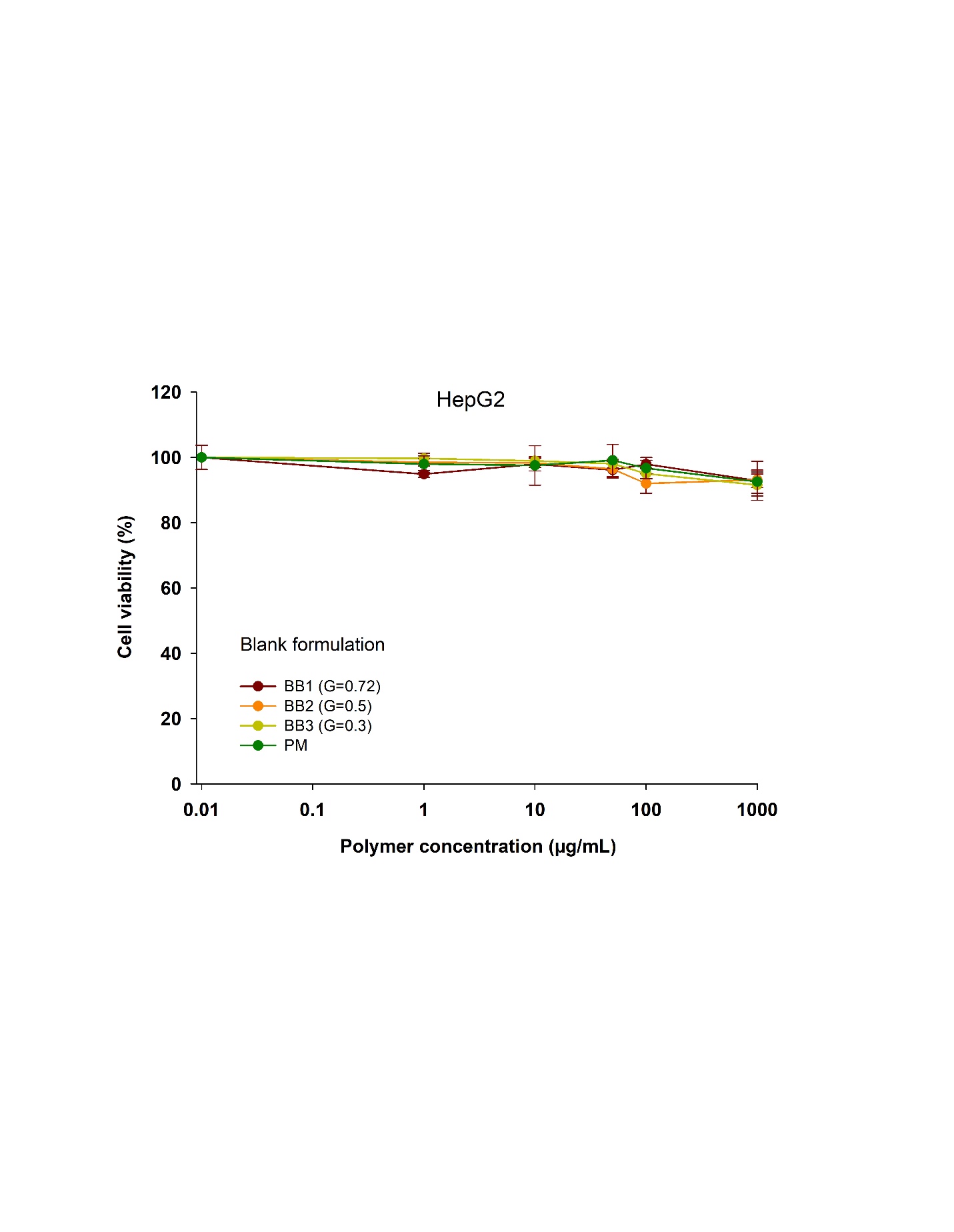


Fig S11. Cells viability after 72h incubation with different concentrations of blank BBs and linear polymer. HepG2 cells.


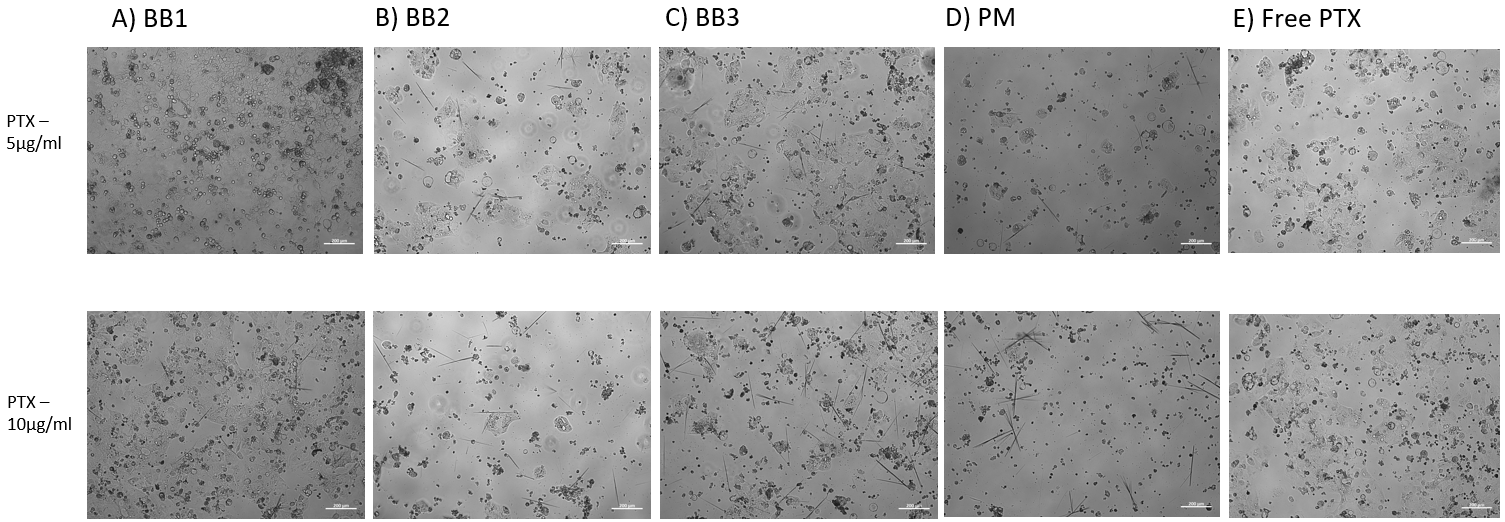


Fig. S12. Microscope images in bright field mode of Caco-2 cells. After being treated with A) BB1, B) BB2, C) BB3, D) PM, and E) Free PTX for 72 hours. Scale bar is 200µm.


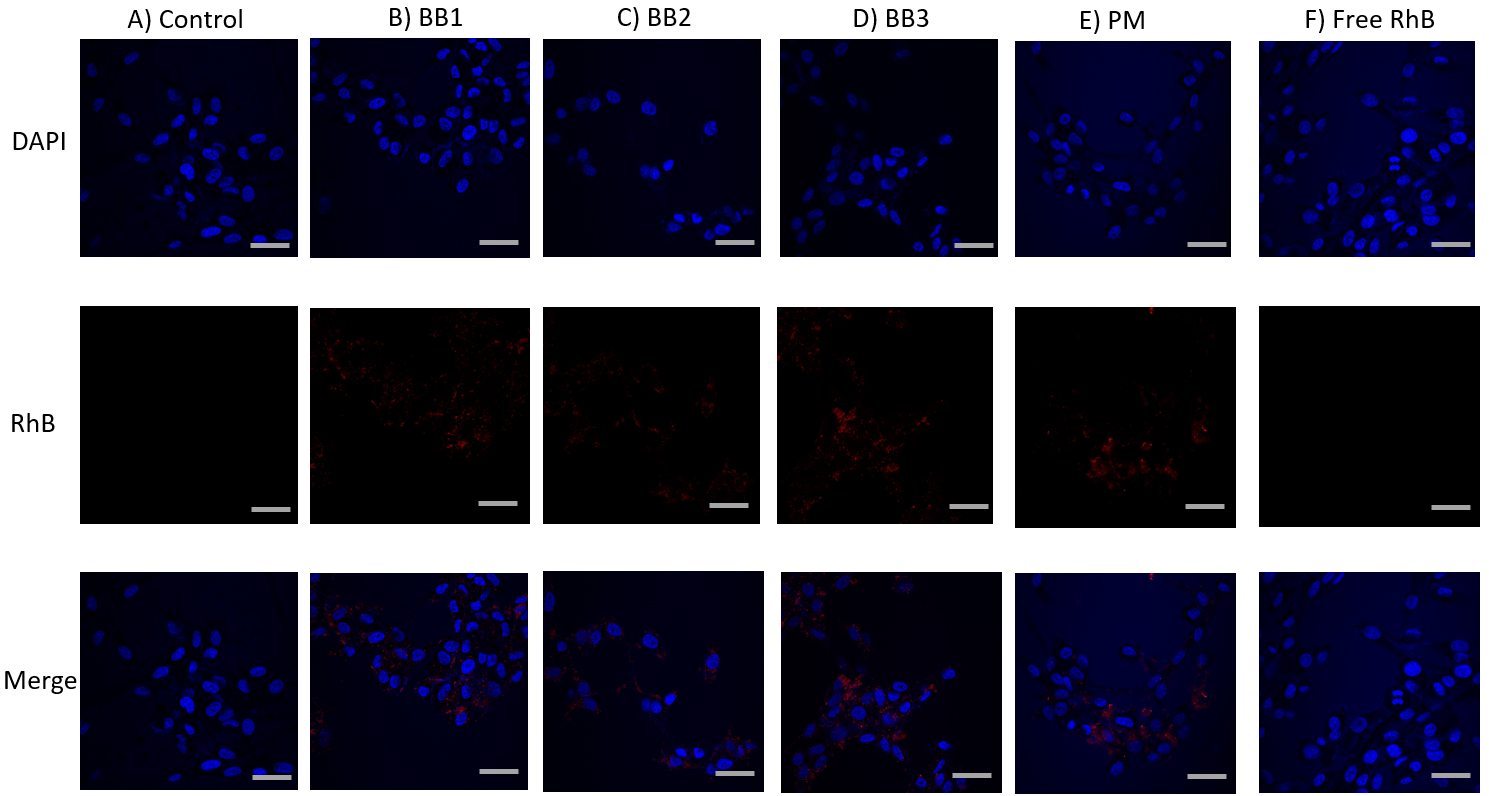


Fig. S13. CLSM images of U87 cancer cell monolayers after 24 h incubation. A) Negative control, B) RhB loaded BB1, C) RhB loaded BB2, D) RhB loaded BB3, E) RhB loaded PM and F) Free RhB. Fluorescence channels are divided into series consisting of fluorescence emitted by cell nuclei with DAPI staining, RhB-loaded BBs/PM and both nuclei and polymeric formulations merged. Scale bars = 100 µm.


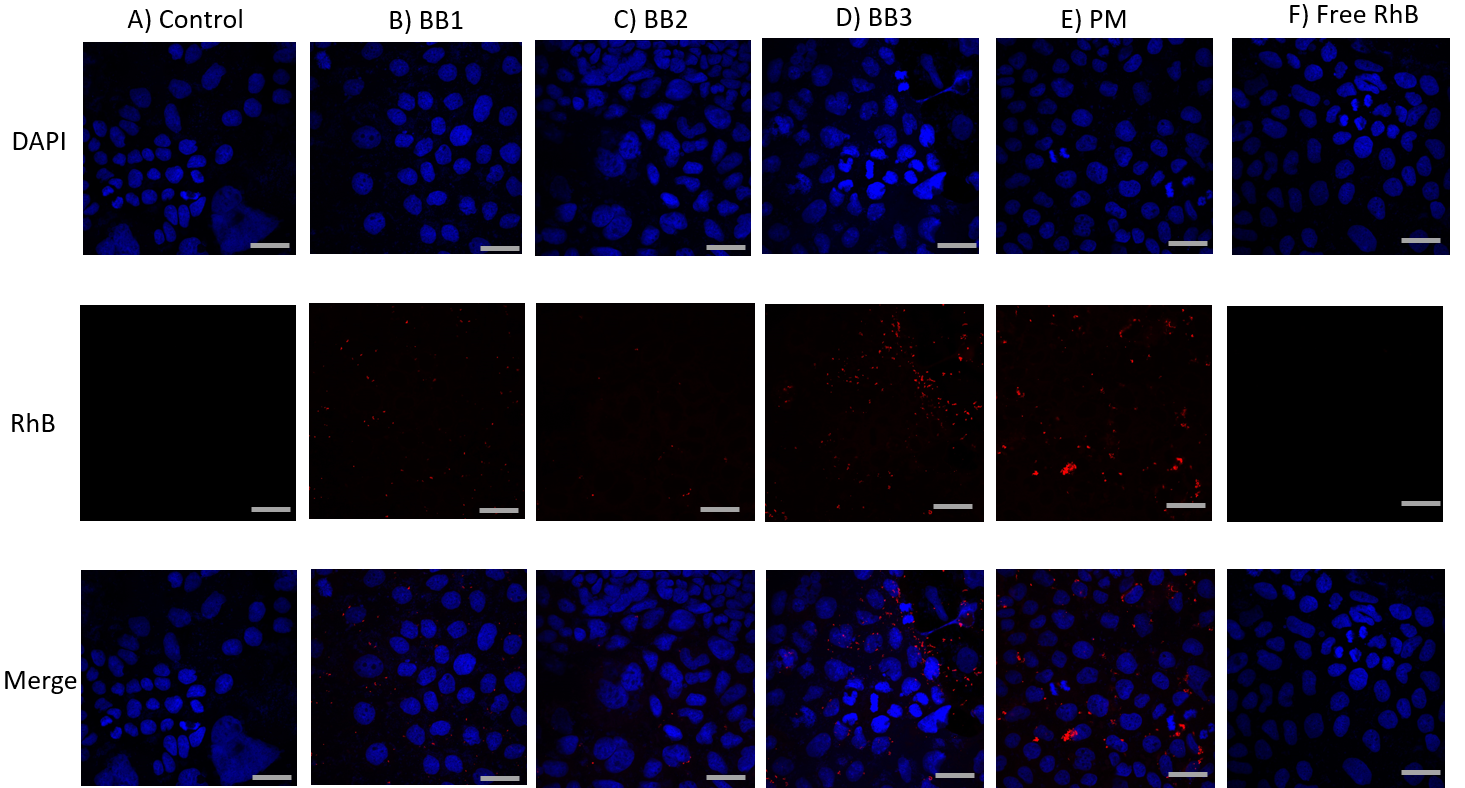


Fig. S14. CLSM images of Caco-2 cancer cell monolayers after 24 h incubation. A) Negative control, B) RhB loaded BB1, C) RhB loaded BB2, D) RhB loaded BB3, E) RhB loaded PM and F) Free RhB. Fluorescence channels are divided into series consisting of fluorescence emitted by cell nuclei with DAPI staining, RhB-loaded BBs/PM and both nuclei and polymeric formulations merged. Scale bars = 100 µm.


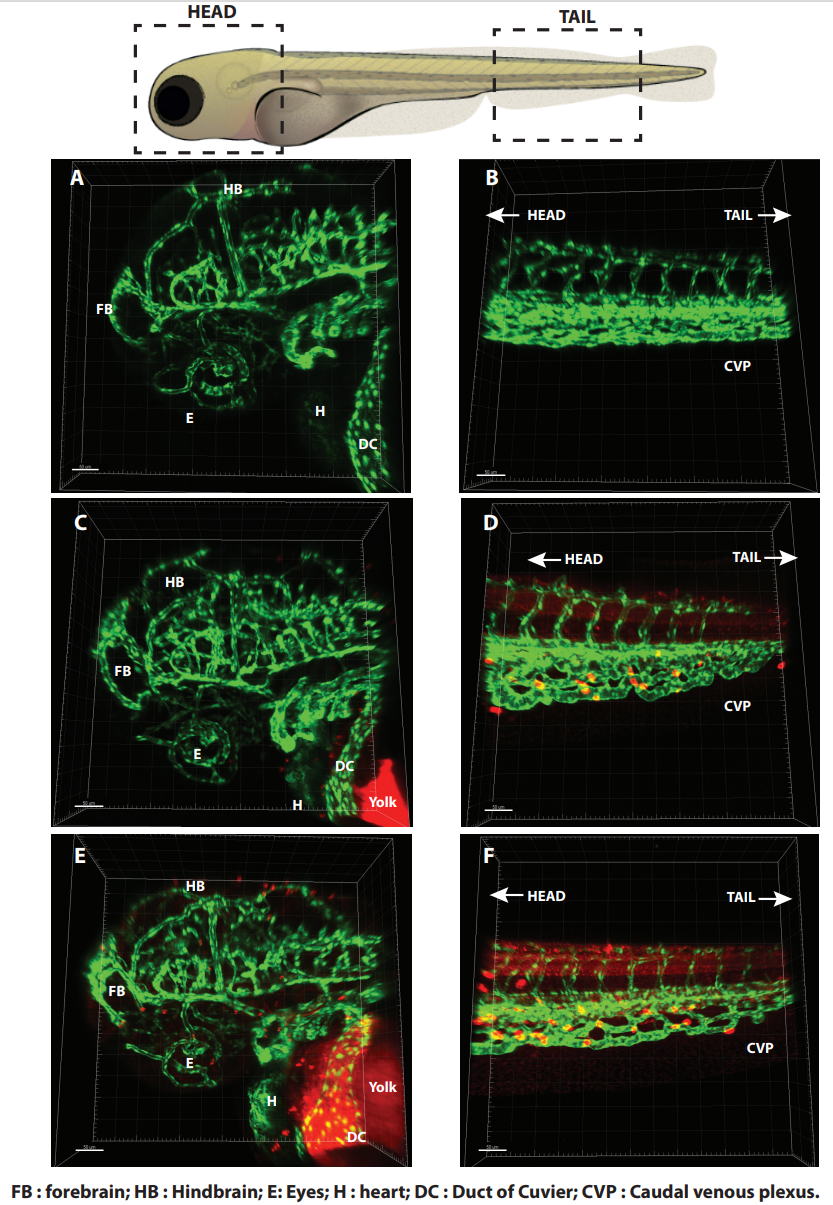


Fig S15. Head and caudal venous plexus confocal images of 50 hpf (flk1:EGFP) transgenic zebrafish larvae. After injection with BB3 or PM, the images were acquired at 2 hours post injection. Images represented are 3D reconstructed images with Imaris® of z-stack acquired as described in Material and methods. A and B: control larvae (no injection); C and D: larvae injected with PM; E and F: larvae injected with BB3 polymer. Red channel: Cy5-labelled PM or Cy5-labelled BB polymer particles; Green channel: EGFP expressed in larvae vascular endothelial cells cytoplasm. Abbreviations: FB: forebrain; HB: Hindbrain; E: Eyes; H: heart; DC : Duct of Cuvier (injection site); CVP : Caudal venous plexus. Scale bar: 100µm

REFERENCE

1. Li, L., et al., *Development of spectral nano-flow cytometry for high-throughput multiparameter analysis of individual biological nanoparticles.* Analytical Chemistry, 2023. 95(6): p. 3423-3433.
